# One-Step Synthesis, Crystallography, and Acute Toxicity of Two Boron–Carbohydrate Adducts That Induce Sedation in Mice

**DOI:** 10.3390/ph17060781

**Published:** 2024-06-14

**Authors:** Ricardo Ivan Cordova-Chávez, José G. Trujillo-Ferrara, Itzia I. Padilla-Martínez, Héctor González-Espinosa, Antonio Abad-García, Eunice D. Farfán-García, Clara Ortega-Camarillo, Alejandra Contreras-Ramos, Marvin A. Soriano-Ursúa

**Affiliations:** 1 Laboratorio de Neurofisiología, Sección de Estudios de Posgrado e Investigación, Escuela Superior de Medicina, Instituto Politécnico Nacional, Plan de San Luis y Díaz Mirón s/n, Mexico City 11340, Mexico; ivancoch@hotmail.com (R.I.C.-C.); gonzalezespinosahector@gmail.com (H.G.-E.); abadantonio19315@gmail.com (A.A.-G.); 2 Laboratorio de Bioquímica, Sección de Estudios de Posgrado e Investigación, Escuela Superior de Medicina, Instituto Politécnico Nacional, Plan de San Luis y Díaz Mirón s/n, Mexico City 11340, Mexico; efarfang@ipn.mx; 3 Laboratorio de Química Supramolecular y Nanociencias, Unidad Profesional Interdisciplinaria de Biotecnología, Instituto Politécnico Nacional, Av. Acueducto s/n, Barrio la Laguna Ticomán, Mexico City 07340, Mexico; ipadillamar@ipn.mx; 4 Medical Research Unit in Biochemistry, Specialties Hospital, National Medical Center SXXI, Instituto Mexicano del Seguro Social, Av. Cuauhtémoc 330, Col. Doctores, Alc. Cuauhtémoc, Mexico City 06720, Mexico; cocamarillo2014@gmail.com; 5 Laboratory of Molecular Biology in the Congenital Malformations Unit, Children’s Hospital of Mexico Federico Gomez (HIMFG), Calle Dr. Marques 162, Col. Doctores, Alc. Cuahutémoc, Mexico City 06720, Mexico; acora_ramos@hotmail.com

**Keywords:** boron, carbohydrate, sedation, acute toxicity, adducts, neurons, mice

## Abstract

Boronic acids form diester bonds with cis-hydroxyl groups in carbohydrates. The formation of these adducts could impair the physical and chemical properties of precursors, even their biological activity. Two carbohydrate derivatives from d-fructose and d-arabinose and phenylboronic acid were synthesized in a straightforward one-step procedure and chemically characterized via spectroscopy and X-ray diffraction crystallography. Additionally, an acute toxicity test was performed to determine their lethal dose 50 (LD_50_) values by using Lorke’s method. Analytical chemistry assays confirmed the formation of adducts by the generation of diester bonds with the β-d-pyranose of carbohydrates, including signals corresponding to the formation of new bonds, such as the stretching of B–O bonds. NMR spectra yielded information about the stereoselectivity in the synthesis reaction: Just one signal was found in the range for the anomeric carbon in the ^13^C NMR spectra of both adducts. The acute toxicity tests showed that the LD_50_ value for both compounds was 1265 mg/kg, while the effective dose 50 (ED_50_) for sedation was 531 mg/kg. However, differences were found in the onset and lapse of sedation. For example, the arabinose derivative induced sedation for more than 48 h at 600 mg/kg, while the fructose derivative induced sedation for less than 6 h at the same dose without the death of the mice. Thus, we report for the first time two boron-containing carbohydrate derivatives inducing sedation after intraperitoneal administration. They are bioactive and highly safe agents. Further biological evaluation is desirable to explore their medical applications.

## 1. Introduction

Boron-containing compounds (BCCs) are interesting molecules in pharmacological sciences because many of them are bioactive compounds, thus being attractive for study as potential drugs [1]. In particular, some boronic acids can easily form diester bonds with molecules with hydroxyls, yielding stable boron-containing adducts [2].

In nature, some BCCs have similar characteristics to those reported herein, such as fructoborate (bis[β-d-fructofuranosato(2-)-κ^2^O^2^,O^3^]borate), formed by the reaction of two fructose molecules and a boron atom, which is synthesized by plants [3]. Some properties are conferred to that molecule, for example, protective activity against metabolic and inflammatory disorders in humans [1]. This is a fact that supports the notion that structurally related compounds can be studied as potential drugs [3]. In animal cells, only compounds presenting cis-diol moieties have been reported as capable of linking to boron-containing moieties [4]. Although the knowledge of the structure–activity relationship of BCCs in animals is poor, it is proposed that, when boron is positioned on a biologically active molecule in an electron donor region, it can generate potent biological activity because of the strong hydrogen and covalent bonds that it is able to create on target proteins [5].

Furthermore, synthetic procedures carried out to form diol–boronic adducts have been explored. In this sense, several adducts derived from phenylboronic acid and carbohydrates, as well as crystals derived from β-fructopyranose [6], α-d-glucofuranose [7], and β-d-arabinopyranose [8], have been reported. The formation of these adducts is interesting, among other reasons, because of the disposition of different hydroxyl groups with the feasibility of reaction. In previously reported structures, the formation of diester bonds has been observed in a stereoselective reaction, while the formation of several different five- or six-ring carbohydrates is also possible [9]. The applications of these structures are yet to be explored; however, they have been studied in the field of biomaterials for the design of sensors [10]. One of the most promising applications as sensors involves the development of self-regulated insulin delivery materials in the control of glycemia of patients living with diabetes mellitus [11,12]. Other types of boron-containing monosaccharides have been designed for the overexpression of glucose transporters in brain tumors because of the increased anaerobic metabolism in cancerous cells [13].

It should be mentioned that there are limited studies on the biological activity and toxicity of some BCCs. However, the safety of some BCCs in mammals allows us to explore their biological activity and potential as drugs [14,15]. An example is phenylboronic acid, the precursor of the three mentioned compounds. It has a lethal dose 50 (LD_50_) of 900 mg/kg with i.p. administration in mice [16], but no acute toxicity of its carbohydrate derivatives has been reported. These facts have led us to evaluate the biological actions, as well as the biological activity, of these derivatives.

However, BCCs [17,18] and some carbohydrate-derived compounds (for example, topiramate and dapagliflozin) have been reported to affect the central nervous system, as they have shown multiple biological actions, such as interactions in calcium channels or on enzymes, as well as inducing several changes, which could be involved in some phenomena in neuronal and metabolism functions [19].

In this work, we describe a straightforward one-step synthesis procedure applied to obtain two carbohydrate-derived BCCs. Moreover, due to the scarce information on the bioactivity of boron-containing carbohydrates, the acute toxicity and sedative effect observed after the intraperitoneal administration of these compounds are characterized.

## 2. Results

### 2.1. Chemistry

**Figure 1:**
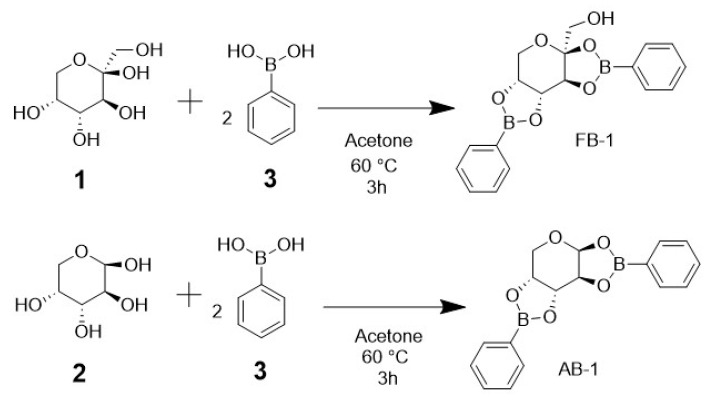
Scheme of the synthesis reaction for the carbohydrate–boron adducts. Each carbohydrate ((**1**) d-fructose or (**2**) d-arabinose) equivalent requires two equivalents of (**3**) phenylboronic acid to generate two diester bonds for the new adducts.

The synthesis of BCC adducts was performed via condensation between sugar and phenylboronic acid in a 1:2 ratio in acetone under very mild conditions. This straightforward one-step synthesis procedure is depicted in Figure 1. Crystals were formed after 2 days; then, all characterization tests were performed. Both the fructophenylborate adduct (**FB-1**) and the arabinophenylborate adduct (**AB-1**) had a transparent, crystalline, and geometric structure, with the formation of crystals of approximately 5 × 2 mm for **FB-1** and of 3 × 1 mm for **AB-1**. The yields were 76% for **FB-1** and 70% for **AB-1**. Regarding solubility, **FB-1** was soluble in alcohols (methanol, ethanol, isopropanol, and butanol), while **AB-1** was not soluble. The compounds were not completely pure; thus, purification was performed to remove the starting material (phenylboronic acid and saccharides). The extraction of saccharides was considerably easy, carried out by means of washing with distilled water. Carbohydrates are water-soluble, whereas **FB-1** and **AB-1** are not. Regarding phenylboronic acid purification, the process for **FB-1** was a major challenge: **FB-1** had the same solubility as phenylboronic acid in almost all the solvents used. It was only soluble in dichloromethane, whereas phenylboronic acid was not, so the dissolved adduct had to pass through a rotavapor in order to evaporate dichloromethane. For AB-1, ethanol was useful because of the insolubility of **AB-1** in alcohols, while phenylboronic acid was completely soluble. In order to recrystallize to obtain crystals suitable for X-ray diffraction, both adducts were dissolved once again in acetone, and the solvent was allowed to slowly evaporate.

In thin-layer chromatography, the Rf value for **FB-1** was 0.42, while the Rf value for AB-1 was 0.49, employing a mobile phase of hexane/ethyl acetate in a 2:3 ratio. There was no evidence indicating that the precursors left a spot in the same line where the products were positioned.

### 2.2. X-ray Diffraction Crystallography

**Figure 2:**
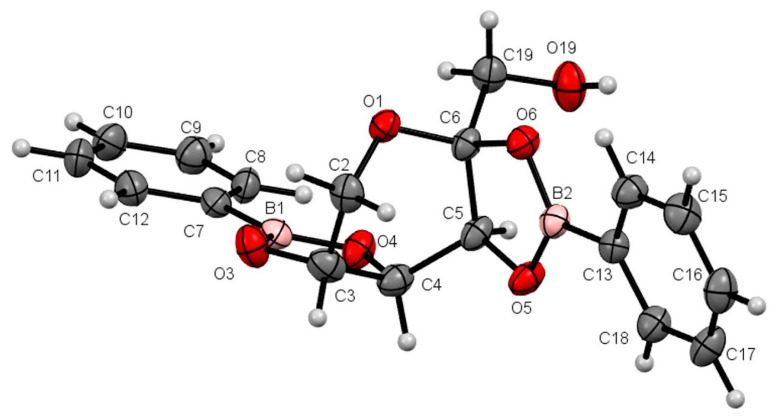
ORTEP at a 30% probability level of β-d-fructopyranoborate (**FB-1**); the acetone molecule is omitted for clarity. Selected lengths (Å): B1–O3, 1.360(4); B1–O4, 1.365(4); B2–O6, 1.368(4); B2–O5, 1.373(4); C5–O5, 1.436(3); C6–O6, 1.439(3); C3–O3, 1.438(3); C4–O4, 1.436(3). Selected bond angles (°): O3–B1–O4, 113.4(3); O6–B2–O5, 112.7(2). Selected torsion angles (°): O3–C3–C4–O4, 12.6(3); O5–C5–C6–O6, 9.2(3).

**Figure 3:**
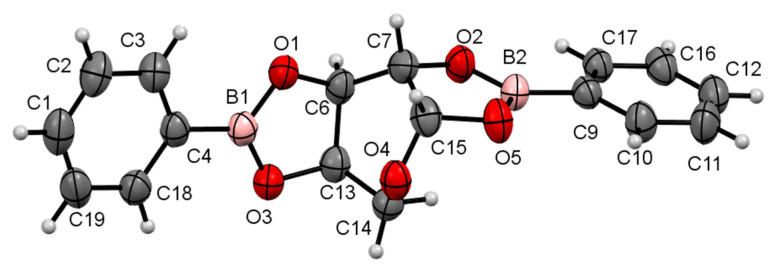
ORTEP at a 30% probability level of β-d-arabinopyranoborate (**AB-1**). Selected lengths (Å): O1–B1, 1.370(8); O1–C6, 1.436(7); O2–B2, 1.371(7); O2–C7, 1.449(7); O3–B1, 1.353(8); O3–C13, 1.448(7); O5–B2, 1.369(8); O5–C15, 1.434(7). Selected bond angles (°): O3–B1–O1, 112.6(5); O5–B2–O2, 112.5(5). Selected torsion angles (°): O2–C7–C15–O5, 17.0(6); O1–C6–C13–O3, 11.4(5).

The compounds were crystallized accurately in this study. The compounds are illustrated in the Oak Ridge Thermal Ellipsoid Plots (ORTEPs) in Figure 2 and Figure 3.

### 2.3. In Silico Predictions

On the Protox II server, the predicted LD_50_ for **AB-1** was 180 mg/kg, with a toxicity class of 3, while for **FB-1,** the predicted LD_50_ was 1000 mg/kg, with a toxicity class of 4 (see Supplementary Materials).

The SwissADME server showed a LogP of 0.33 for **AB-1**, and the Egan graphic showed high absorption in the gastrointestinal tract. For **FB-1**, a LogP of −0.30 was shown, and the Egan graphic showed high absorption in the gastrointestinal tract.

The Molinspiration server predicted a LogP of 2.26 for **AB-1** and a TSPA value of 46.17. For **FB-1**, a LogP of 1.7 and a TSPA value of 66.4 were shown. Both servers predicted no violation of the Lipinski rule of 5 for the two compounds.

The SwissTarget server produced the following results: For **AB-1**, there was a 33.3% chance of binding to a Family A G-protein-coupled receptor and a 33.3% chance of binding to enzymes. The most related targets were type II, I, XII, and IX carbonic anhydrases; the phenylalanyl-tRNA synthetase mitochondrial; the estrogen receptor beta; A1, A2a, A2b, and A3 receptors; the endothelium receptor ET-A; hexokinase type IV; alkaline phosphatase; tissue-nonspecific isoenzymes; the bifunctional protein NCOAT; and arachidonate 15-lipoxygenase. For **FB-1**, there was a 33.3% chance of binding to a Family A G-protein-coupled receptor, a 13.3% chance of binding to electrochemical transporters, and a 13.3% chance of binding to proteases. The main targets were A1, A2a, A3, and A2b adenosine receptors; ADAM17; the bifunctional protein NCOAT; hexokinase type IV; transmembrane domain-containing protein TMIGD3; protein kinase C alpha; thymidine kinase; butyrylcholinesterase; matrix metalloproteinase 13; and sodium/glucose cotransporter types II and I.

### 2.4. Acute Toxicity Test

In the first phase of the acute toxicity test using Lorke’s method, three mice were employed for each of the three doses. For both **FB-1** and **AB-1**, all the mice in the three groups survived, despite the dose applied (10, 100, and 1000 mg/kg body weight).

For the second phase, a single 1600 mg/kg BW i.p. dose for each of the products was administered (using three mice for each compound), and the two animals of both groups did not survive. The geometric mean was calculated between the doses of 1000 mg/kg (the greatest dose at which all the mice survived) and 1600 mg/kg (the smallest dose at which all the mice died), obtaining an LD_50_ value of 1265 mg/kg for both **FB-1** and **AB-1**.

### 2.5. Determination of the Median Effective Dose (ED_50_) for Hypnosis and Sedation

In the first phase of the acute toxicity test, it was observed that both mice administered the 1000 mg/kg dose of **FB-1** and **AB-1** fell asleep, while the mice administered the 10 and 100 mg/kg doses had no behavioral impairment. The doses established for the second phase were 140, 225, 370, 470, and 600 mg/kg, and each compound was applied at these doses to three mice.

In both groups, hypnosis signs were observed at the 470 mg/kg dose. The ED_50_ for hypnosis was calculated using probit curves, resulting in a dose of 417 mg/kg.

As observed in the hypnosis test, the sedation test had similar results for both compounds studied. Sedation signs were observed at the 600 mg/kg dose. The estimated ED_50_ for sedation was 531 mg/kg.

**Figure 4:**
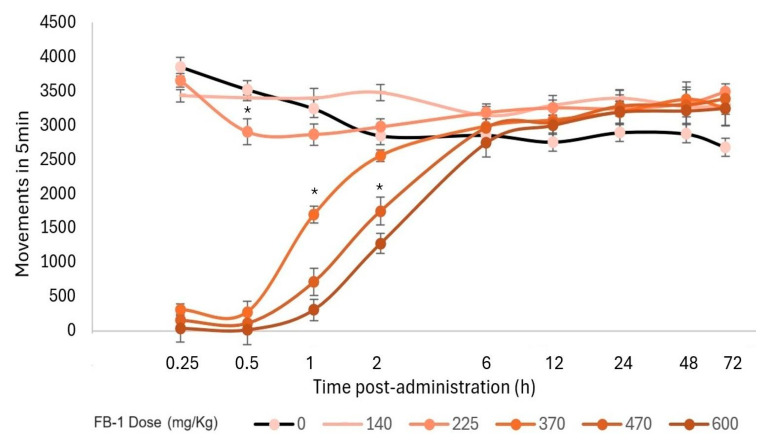
Motor performance in the open-field test. Motor activity that occurred within 5 min and the effect on locomotion after **FB-1** administration. The markers represent the mean, while the bars represent the standard error of the mean. Asterisks indicate the lowest dose, which, at each measured time, reduced the total movements compared to the control group, *p* < 0.05; *n* ≥ 4.

**Figure 5:**
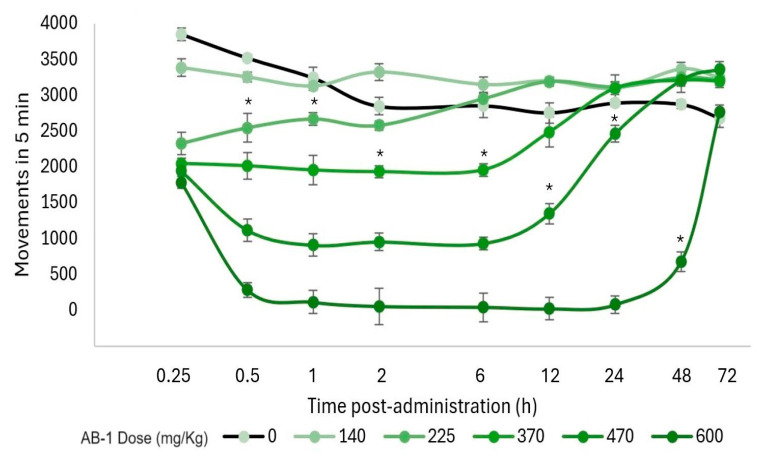
Motor performance in the open-field test. Motor activity that occurred within 5 min and the effect on locomotion after **AB-1** administration. The markers represent the mean, while the bars represent the standard error of the mean. Asterisks indicate the lowest dose, which, at each measured time, reduced the total movements compared with the control group, *p* < 0.05; *n* ≥ 4.

**Figure 6:**
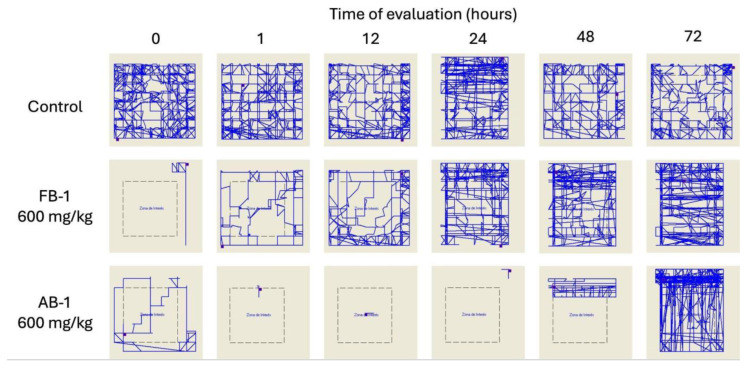
Motor performance in the open-field test. Illustrative reports of the effects of the administration of FB-1 or AB-1 (in the highest tested doses).

Additionally, a motor evaluation was performed as a complement to the visual evaluation of sedation and hypnosis because it allowed for the observation of diminished activity after **FB-1** or **AB-1** administration (Figure 4, Figure 5 and Figure 6). As notable in the plots, **FB-1** administration (at doses of ≥370 mg/kg) induced sedation and diminished motor activity before the first hour with recovery at 6 h, while **AB-1** administration (at doses of ≥225 mg/kg) induced sedation and diminished motor activity before the first hour with recovery at 6 to 72 h (dose-dependent).

## 3. Discussion

Two BCCs, one derived from d-fructose and the other from d-arabinose, both with phenylboronic acid as the boron compound precursor, were synthesized, and their chemico-biological profiles were extensively analyzed for the first time. The synthesis reaction allowed for a yield higher than 70%, as well as reducing the waste of the reagents employed. Although the method used to synthesize the boron–carbohydrate adducts in our laboratory was different from the procedures previously used by other authors to obtain these compounds (in different reports) [6,8], mainly in terms of the use of different solvents and recrystallization techniques, the products obtained had the same macroscopic characteristics.

Thin-layer chromatography tests showed that the products obtained had Rf values different from the carbohydrates (as well as phenylboronic acid, supporting the formation of new products). The melting point for the products was different from that reported for the used reagents. It has been reported that some borates are capable of transferring hydrophilic molecules (such as saccharides) across the cell membrane because of their high lipophilicity [20,21,22].

The infrared and Raman spectra were complementary, and characteristic signals were observed. In **FB-1**, the stretching of O–H bonds was observed at a wavelength of 3400 cm^−1^, C-H was observed at 3000 cm^−1^, signals corresponding to B–O bonds were observed at 1500 cm^−1^, and signals of the phenyl group were observed at 1300 cm^−1^. All of these signals were also present in the Raman and infrared spectra for **AB-1**, with the exception of the O–H stretching signal at 3400 cm^−1^; this is justified by the fact that, with the formation of diester bonds, new B–O bonds are formed, so the new compound has no hydroxyl groups. Thus, the signal for this structure was missing, which was expected.

The ^1^H NMR spectra of both compounds showed signals in the range of 7–8 ppm, corresponding to the protons in the aromatic rings. The most important signals were those involving the carbon atoms where the diester bonds were formed. The anomeric carbon of the carbohydrate ring in **FB-1** had no proton, so it did not produce any signal by itself. The proton of the carbon in position 2 had one vicinal proton in the *trans* position, with a *J* value of 3.6 Hz and a double signal, and it appeared in δ4.31. The proton linked to the carbon of position 3 had two different vicinal protons, one in the *trans* position and the other in the *cis* position, resulting in two different *J* values of 5.9 Hz and 2.6 Hz, with a double doublet because of the unfolding signals. The proton in the carbon of position 4 had one vicinal proton in the *cis* position, but it was linked to a methylene group, with two more protons in this position, resulting in a triple doublet signal with *J* values of 9.1 Hz and 4.7 Hz.

The ^1^H NMR spectra of **AB-1** had a special feature because it lacked the hydroxymethyl present in the previous compound (fructose is a hexose, while arabinose is a pentose), so the anomeric carbon had a proton, and it produced a signal in this spectrum; this was a double signal, and the vicinal proton was in the *cis* position, so the *J* value was 5.0 Hz. Interestingly, the proton linked to the carbon in position 2 now had two vicinal protons with different *J* values because one proton was in the *trans* position, while the other one was in the *cis* position, and the unfolding signals caused by the two different *J* values generated a double doublet. This could only be possible if d-arabinose formed a ring in the β-anomer because this allows for the oxygen linked to the carbons in positions 1 and 2 to be placed in the same plane, so the reaction for the formation of the diester bond occurs in the *cis* position (it should be noted that diester bonds are not possible if the oxygens are in the *trans* position). With the Karplus equation, we could confirm, using the *Φ* torsion angles, the *J* values of the *cis* and *trans* dispositions of the protons involved [23]. The vicinal protons with a *cis* position had an angle from 0 to 90° with a lower *J* value (2.5–3.2 Hz), while the *trans* vicinal protons had a 90-to-180° torsion angle, with a greater *J* value (5.0 ± 1 Hz) [24].

If the carbohydrate had closed itself in an α anomer, one of the two diester bonds formed in the new adducts would not be formed. When monosaccharides are dissolved in an aqueous solution, they form different isomers with six- and five-membered rings with α and β anomers. Some carbohydrates such as fructose are more likely to bind to boronic acids than others such as glucose because the fraction of isomers with syn hydroxyl groups is higher in fructose (nearly 25%) than in glucose (1%) [25].

Regarding the formation of 1,3- and 1,4-diols cyclic boronic esters with five-, six-, or seven-membered rings, with enhanced N–B interactions, several authors established the mechanisms by which boronic acids are able to bind some saccharides, and, once linked, the pH decrease in these compounds modulates the fluorescence of certain fluorophores, making the identification of specific carbohydrates possible [26,27,28,29]. However, those studies mentioned the effect on N–B bonds exerted by the interaction of BCCs with molecules that can form 1,3- and 1,4-diols. Nevertheless, the unique interaction reported in the binding to carbohydrates takes place with furanoses, such as d-fructofuranose, where boronic acid generates 2,3-*cis*-diol with furanose, but 4,5-diol is not formed because the hydroxyl groups are in the *trans* position [30]. With the analysis of this work, we propose that the cyclization of d-fructopyranose and d-arabinopyranose allows for the formation of two diols for each saccharide molecule.

The ^13^C NMR spectra provided relevant information allowing for the confirmation of the presence of a specific anomer formed in the synthesis reaction. In the 90–100 ppm range of the ^13^C NMR spectrum, signals corresponding to the anomeric carbon, α or β, were observed [31]. In the case of both carbohydrate-derived boron adducts, just one signal was observed in the abovementioned range, which means that only one anomer formed after the synthesis reaction. On its own, this result does not indicate whether the anomer formed in the synthesis reaction was α or β; however, the ^1^H NMR spectra previously analyzed indirectly prove the cyclization as a β-anomer.

It was possible to conduct X-ray diffraction on both compounds because the crystals obtained had an accurate crystalline and geometric appearance allowing for suitable diffraction for this technique. This study confirmed what was observed using the previously mentioned spectroscopy techniques: The new carbohydrate-derived boron compounds formed via the cyclization of the carbohydrate in β-pyranose, and they formed two diester bonds with two molecules of phenylboronic acid, namely, 2,3-*cis*-diol and 4,5-*cis*-diol for **FB-1** and 1,2-*cis*-diol and 3,4-*cis*-diol for **AB-1**. The position of the two oxygens in each diester bond was in the same plane, so the reaction was in the *cis* position.

The d-fructose equilibrium in water showed that the pyranose form was the most favorable when cycling, with a proportion of 72%, while the proportion of furanose was 28%; in the β-anomer, the proportion of pyranose was 70%, and the proportion of furanose was 23%, being the predominant anomer [32]. For the formation of **FB-1**, the most predominant isomer, β-fructopyranose, is ideal because it allows for the binding of two equivalents of phenylboronic acid, forming two diester bonds. There is not enough information about the isomer forms of d-arabinose in aqueous solutions, but it is well described in L-arabinose that the α-anomer has a higher proportion in nonpolar solvents, obeying the anomeric effect, while cyclization can be variable depending on the solvent, being up to 33% in dimethyl sulfoxide and only 3% in deuterated water in the furanose form. At least with these two solvents, it seems that the pyranose form tends to be the dominant proportion [33]. The acetone used in the synthesis was a polar solvent; therefore, it was expected that a higher proportion of the β-anomer would be found, going against what the anomeric effect dictates for both compounds.

It is possible that the α-anomer could also be formed in the synthesis reaction but that the proportion was not enough to generate any significant signal. The absence of the anomeric effect could explain this phenomenon: It is possible that more than 95% of the newly synthesized compound closed as a β-anomer. Nevertheless, once the β-anomer was used, the proportion of the α-anomer was higher, so an interconversion of anomers occurred to maintain the proportion of 95% β-anomer and 5% α-anomer. Therefore, the anomeric effect did not occur in this reaction, as expected for a hexopyranose, just like what was observed with the glucose.

Regarding the biological activity of boron–carbohydrate adducts, the information is limited. In fact, only a few reports have suggested their role as potential labels or agents modifying metabolism [19], and there are no reports on the toxicity of the BCCs in the current study. In this study, it was observed that, at a dose of 1000 mg/kg, the mice were sedated 5 min after the administration of **FB-1**, and this effect lasted for about 2 h, while for **AB-1,** it lasted for 72 h after administration. After that time, the mice could recover completely, and they had no neurological or motor complications. At a dose of 1600 mg/kg body weight, the mice were sedated within the first 3–5 min after administration. However, they did not survive longer than 5 to 6 h. As both compounds have an LD_50_ higher than 1200 mg/kg, they can be declared safe for administration. It is intriguing that phenylboronic acid has an LD_50_ of 900 mg/kg, considerably lower than that calculated for the adducts, but as it is the precursor of these products, and its structure is almost intact, it presents less acute toxicity. The two hydroxyl groups in phenylboronic acid were replaced by cys-diol unions with saccharides. Both the saccharide and the borate precursor lost their most polar component (the hydroxyl groups), so the new compounds were significantly more nonpolar (**FB-1** had just one hydroxymethyl, while **AB-1** completely lacked hydroxyls). Therefore, due to their lipophilic profile, they have an increased chance of crossing the blood–brain barrier (BBB). This is related to the triggered hypnosis and sedation, both of which are neurological effects.

The hypnosis and sedation effects observed in both compounds led us to review the properties of the compounds and the implications in a therapeutic manner: the study of the ED_50_ is the beginning of this extension of the protocol, aiming to establish safe doses for future studies on potential new drugs [34].

The mechanism of action is unclear; however, in silico predictors suggest that compounds with carbohydrate-like structures and some BCCs reported as sedatives act on membrane channels. Among these, some well-known benzodiazepines, such as alprazolam, have been probed to form complexes with boric acid, and boronic acids have been crystallized [35]. In fact, alprazolam works by binding to the GABA-A receptor, increasing the activity of γ-aminobutyric acid (GABA), an inhibitory neurotransmitter [36]. However, boric acid was named “sedative salt” after being isolated from borax by Wilhelm Holmberg in 1702 [37]. Nonetheless, this sedative salt was observed to have a toxic effect, resulting in the death of various people, mainly children, which is why its use for such purposes was stopped, and its mechanism of action remained unestablished [38,39,40,41,42].

Another drug with a possible effect on GABAergic activity is topiramate [43], whose structure mainly comprises the six-membered ring of fructose [44]. However, several mechanisms of action have been described for topiramate’s antiepileptic effects. Additional mechanisms include the blockage of voltage-dependent sodium channels [45], a negative modulatory effect on L-type calcium channels [46], the inhibition of carbonic anhydrase isoforms [47], and the antagonism of the *N*-methyl-d-aspartate glutamate receptor [48]. It should be noted that the structure of topiramate is clearly similar to that of the adducts presented in this work, particularly that of **FB-1**; thus, it is hypothesized that the similarity in their structures confers them with similarity in their biological activities, and maybe they share protein targets.

Even though both synthetic compounds had the same ED_50_ for hypnosis and sedation, the behavioral effects observed were significantly different. For **FB-1**, the behavior impairment began 5 min after administration, while **AB-1** triggered the effects 10 to 15 min later. The most important finding was the duration of the sedative effects: **FB-1** had a hypnosis effect that lasted for about 60 min and a sedative effect that lasted for 2 h, while **AB-1** had a hypnosis effect that lasted for more than 5 h and a sedative effect that lasted for up to 72 h. The animals had to be strictly observed because they could not feed by themselves due to the prolonged effect. In order to avoid death from starvation, they were fed by the lab personnel until their full recovery.

The limitations of our study include the requirement to use 5% DMSO solution as a vehicle due to the poor solubilization of the compounds in water or saline solution. Furthermore, intraperitoneal administration was carried out, while oral administration would allow for a better comparison to other well-known sedative drugs, such as topiramate.

Another limitation concerns the clear elucidation of the mechanism of action; although topiramate and the tested adducts have high structural homology, topiramate has multiple mechanisms of action. Moreover, there are other possible mechanisms by which this phenomenon occurs. For example, the kinetic behavior of adducts could release sugars, phenylboronic acid, or other unknown metabolites, thereby inducing the observed biological effects.

Further studies are required to elucidate the mechanism(s) of action and whether the pharmacodynamic and pharmacokinetic profiles of these two compounds are related to the observed induced changes. Further studies are also required to study potential applications in neurological disorders. In addition, it would be interesting to study these compounds under mammalian physiological conditions (temperature and pH) not only to propose applications in their pathologies but also to determine whether their biotransformation could release bioactive compounds and modify carbohydrate metabolism.

## 4. Materials and Methods

### 4.1. Materials

#### 4.1.1. Chemicals

d-(-)-Fructose (CAS 57-48-7), d-(-)-arabinose (CAS 10323-20-3), phenylboronic acid (CAS 98-80-6), acetone (CAS 67-64-1), dimethyl sulfoxide (CAS 67-68-5), dimethylformamide (CAS 68-12-2), acetic acid (CAS 64-19-7), ethanol (CAS 64-17-5), methanol (CAS 67-56-1), butanol (CAS 71-36-3), isopropanol (CAS 67-63-0), ethyl acetate (CAS 141-78-6), chloroform (CAS 67-66-3), dichloromethane (CAS 75-09-2), xylene (CAS 1330-20-7), toluene (CAS 108-88-3), acetaldehyde (CAS 75-07-0), tetrahydrofuran (CAS 109-99-9), carbon tetrachloride (CAS 32488-50-9), acetonitrile (CAS 75-05-8), diethyl ether (CAS 60-29-7), hexane (CAS 110-54-3), and water (CAS 7732-18-5) were obtained from Sigma-Aldrich, Merck (St. Louis, MO, USA). For thin-layer chromatography, the plates used were made from TLC silica gel F_254_ aluminum sheets (Merck, Darmstadt, Germany).

#### 4.1.2. Animals

For acute toxicity tests, 27 male CD1 mice aged 6–7 weeks were employed to examine each compound, for a total of 54 mice. They were obtained from the Vivarium of the Autonomous University of the State of Hidalgo (Universidad Autónoma del Estado de Hidalgo, UAEH). The animals were used according to the specifications of the Mexican Official Norm NOM-062-ZOO-1999-SAGARPA, Technic Specifications for the Production, Care, and Use of Laboratory Animals. The protocol was approved by the Biosecurity Committee of the Superior School of Medicine of the National Polytechnic Institute (Escuela Superior de Medicina del Instituto Politécnico Nacional, ESM-IPN), with approval code ESM-CBS-01/05-01-2022, 2.0. The animals were placed in cages with dimensions of 43 × 53 × 20 cm, with a maximum of 6 mice per cage, at room temperature and with filtered water and rodent food ad libitum. Prior to the study, the mice were acclimatized for one week.

### 4.2. Synthesis and Chemical Characterization

Solubility tests were performed with different solvents, from polar to nonpolar agents. Thin-layer chromatography (TLC) was performed to test the reaction process and as a purity criterion. For this test, a mobile phase of hexane/ethyl acetate in a 2:3 ratio was employed. The silica gel TLC plates were cut into a 3 × 5 cm size; the spots of the compounds to be tested were applied 5 mm above the bottom of the plate, while the limit for the displacement of the mobile phase was 5 mm below the top of the plate. Once the mobile phase was completely dried from the plate, it was analyzed with ultraviolet light to observe the displacement of the precursors and the adducts. Then, the Rf value was calculated.

The melting point was measured using a manual electrothermal fusiometer in a triplicate assay. Infrared spectra were obtained using an FT-IR Perkin Elmer Frontier spectrometer and a UATR polarizer, and they are reported as the wave number of absorption (cm^−1^). Raman spectroscopy was carried out by placing a sample on a quartz slide, which was then placed on a Raman-enhanced microscope (Bruker-Senterra^®^ system, Optik GmbH, Tuerkenfeld, Germany). The spectra were obtained using a laser source of 785 nm, 50 mW power, an integration time of 5 s, and three acquisitions. The resolution was 3–5 cm^−1^, with a spectral range from 1800 to 440 cm^−1^ and an aperture of 50 × 100 µm in a 50× objective.

^1^H, ^13^C, and ^11^B nuclear magnetic resonance spectroscopy was carried out using a Varian Mercury spectrometer operating at 300 MHz, using DMSO-*d*_6_ as a solvent. Chemical shifts (δ) are reported in parts per million (ppm) from the residual solvent peak used as a reference [49], and the coupling constants (*J*) are in Hz. The following abbreviations were used to indicate the multiplicity: s, singlet (s); d, doublet; t, triplet; and m, multiplet. X-ray diffraction crystallography was performed using a Bruker APEXII CCD X-ray diffractometer. Mass spectrometry was performed using a JEOL AccuTOF: JMS-T100LC instrument (Peabody, MA, USA) with the DART^+^ ionization mode.

β-d-Fructopyranobisborate (**FB-1**): ((3a*R*,5a*R*,8a*R*,8b*S*)-2,7-diphenyltetrahydro-3a*H*-bis([1,3,2]dioxaborolo)[4,5-b:4′,5′-d]pyran-3a-yl)methanol: In a round flask, 1 g (5.55 mmol) of d-(-)-fructose, 1.353 g (11.1 mmol) of phenylboronic acid (1:2 ratio), and 40 mL of acetone were placed. The mixture was stirred and heated to reflux for 180 min, monitored with samples of a silica plaque. The resulting solution was filtered and crystallized via nucleation, changing the cooling rate. Crystals were observed after 2 days to obtain 1.498 g (4.26 mmol) of **FB-1** in a 76.8% yield as a white powder, M.P. 94 ± 1 °C; IR ν_max_ (cm^−1^): 1028, 1219, 1499, 1603, 1739, 2971, 3384. Raman shift (cm^−1^): 1001, 1577, 1604, 3064, 3347. ^1^H NMR (300 MHz, DMSO-d6) δ 7.72 (dd, *J* = 12.9, 6.5, 28H), 7.48 (dd, *J* = 15.4, 8.0, 13H), 7.43–7.28 (m, 30H), 5.13 (s, 1H), 4.47 (dt, *J* = 9.1, 4.7, 1H), 4.36 (dd, *J* = 5.9, 2.6, 1H), 4.31 (d, *J* = 3.6, 1H), 4.22 (dd, *J* = 2.9, 2.2, 1H3), 3.92 (dd, *J* = 8.9, 3.3, 1H), 3.40 (dd, *J* = 12.1, 1.8, 1H), 3.24 (dd, *J* = 11.1, 5.2, 1H). ^13^C NMR (75 MHz, DMSO-*d*_6_) δ 135.05 (C17, C21), 134.52 (C22, C26), 132.33 (C12, C16), 128.52 (C18, C20), 128.49 (C20), 127.86 (C23), 127.78 (C25), 97.17 (C4), 72.62 (C6), 72.15 (C5), 72.12 (C1), 72.09 (C2), 60.26 (C10). ^11^B NMR (96 MHz) δ 33.64. MS *m*/*z* 370 [M+H20]^+^. See Supplementary Materials for spectra.

β-d-Arabinopyranobisborate (**AB-1**): (3a*R*,5a*R*,8a*R*,8b*S*)-2,7-diphenyltetrahydro-5H-bis([1,3,2]dioxaborolo)[4,5-b:4′,5′-d]pyran: This was synthesized in the same way described for FB-1 but starting with 0.833 g of d-(-)-arabinose (5.55 mmol), 1.353 g of phenylboronic acid (11.1 mmol), and 40 mL of acetone to obtain 1.251 g (3.89 mmol) of AB-1 in a 70.1% yield as a white powder, M.P. 160 ± 1 °C; IR ν_max_ (cm^−1^): 1026, 1218, 1497, 1602, 1739, 2971. Raman shift (cm^−1^): 998, 1574, 1605, 3057, 3406. ^1^H NMR (300 MHz, DMSO-*d*_6_) δ 7.79–7.69 (m, 9H), 7.56–7.43 (m, 5H), 7.42–7.32 (m, 9H), 5.32 (d, *J* = 1.8, 1H), 4.71 (d, *J* = 2.6, 2H), 4.49 (d, *J* = 2.4, 1H), 4.26 (d, *J* = 10.1, 3H), 3.74 (dd, *J* = 11.6, 3.4 Hz, 2H), 3.37 (dd, *J* = 9.8, 2.4, 2H). ^13^C NMR (75 MHz, DMSO-*d*_6_) δ 135.20 (C7, 14), 133.96 (C15, C19, C20, C24), 132.47 (C17), 131.42 (C22), 128.46 (C16, C18), 128.07 (C21, C23), 115.23 (C9), 86.22 (C10), 74.99 (C2), 74.52 (C1), 63.19 (C6). ^11^B NMR (96 MHz) δ 29.02. MS *m*/*z* 323 [M+H]^+^. See Supplementary Materials for spectra.

### 4.3. Single-Crystal X-ray Molecular Structure

Single crystals of **FB-1** and **AB-1**, suitable for X-ray diffraction, were obtained from saturated solutions of acetone. **FB-1** crystallized with one molecule of acetone. The monocrystal data of the BCCs were recorded on a Bruker D8 VENTURE diffractometer using a graphite monochromator (Mo Kα, λ = 0.71073 Å) at 185(2) K. SAINT software [50] and SORTAV software [51] were used to carry out cell refinement and data reduction, respectively. The structures were solved using direct methods and the SHELXS-97 program [52] of the WINGX package [53]. The full-matrix least-square methods were used to perform the final refinement with the SHELX97 program [52]. The H atoms on C, N, and O were geometrically positioned and treated as riding atoms with C–H = 0.93–0.98 Å, Uiso(H) = 1.2 eq(C) for aromatic carbon atoms. Figures for publication were prepared with Platon [54] and Mercury-CSD [55]. The general crystallographic data for compounds **FB-1** and **AB-1** were deposited in the Cambridge Crystallographic Data Centre (CCDC) as supplementary publication numbers 2351428 and 2351427, respectively, while the CIF files are provided in Supplementary Materials.

### 4.4. In Silico Prediction of Physicochemical Properties

Given the fact that these compounds had not yet been tested, we made use of several online servers, such as Protox II [56], SwissADME [57], SwissTarget [58], and Molinspiration [59], to predict some of their physicochemical properties, as well as their putative targets [60,61].

### 4.5. Biological Evaluation

For acute toxicity tests, a modified version of Lorke’s method was employed [62]. In brief, in the first phase, three groups of mice were established, with a value of *n* = 3, with different doses, namely, 10, 100, and 1000 mg/kg body weight, administered intraperitoneally in a single dose. The second phase involved the formation of five different groups with *n* = 3 using new doses, depending on the results of the first phase. The doses were 140, 225, 370, 600, and 1600 mg/kg body weight, administered intraperitoneally in a single dose. The median LD_50_ was obtained by calculating the geometric mean between the dose value where none of the mice survived and the last dose where all the mice lived.

Additionally, as preliminary tests showed that each compound had a sedative effect when administered at doses higher than 100 mg/kg, a test was set up to define the median effective dose (ED_50_) required to induce hypnosis and sedation. The same scheme from the second phase of Lorke’s method was used for this purpose. Five different doses were administered to the animals. The doses established were 140, 225, 370, 470, and 600 mg/kg body weight, administered intraperitoneally in a single dose. The animals were examined for the first 2 h and then 6, 12, 24, 48, and 72 h after the administration of the compounds. A visual evaluation of the behavior of the mice was carried out to determine whether they presented hypnosis or sedation, as described by Bin et al. [63]. Hypnosis is indicated when righting (recovery from being positioned on the side) takes more than 10 s but less than 60 s; sedation is indicated when righting takes more than 60 s or, by default, sleep. The analysis of this test was carried out with probit and logit curves.

An open-field test was carried out 5 min before the treatments to eliminate the effects of motor disturbance on performance. In brief, the mice were placed into motor activity measuring cages (50 × 50 × 50 cm, with detectors each 2.5 cm, OA-BioMed OMNIALVA^®^, Mexico City, Mexico) to determine the total number of movements, distance traveled, highest speed, and vertical movements as in [64]. This evaluation was repeated at 0.25 0.5, 1, 2, 6, 12, 24, 48, and 72 h after treatment.

## 5. Conclusions

Two organoboron–carbohydrate derivatives were obtained using a simple one-step procedure, and their chemico-biological properties were widely characterized. Diester bonds were formed with two hydroxyls in the *cis* position. The stereoselective reaction was demonstrated using different spectroscopy techniques, as well as X-ray diffraction crystallography.

For the first time, biological action was measured in these BCCs. Thus, it was found that the compounds are less toxic than their boron-containing precursor (phenylboronic acid), with both compounds having an estimated LD_50_ of 1265 mg/kg. Notably, sedative action was observed after their administration, being effective at doses higher than 100 mg/kg. The sedative effect was dose-dependent in both cases, and the onset and lapse of sedation were different between the two: for the FB-1 compound, sedation occurred 30 min after administration and was observed for up to 2 h, while for the AB-1 compound, sedation occurred after the first hour of administration and was observed for up to 72 h.

Additional approaches are required to explore the mechanisms of action and pharmacokinetics of these BCCs.

## Data Availability

Any complementary data of the reported data are available upon request from the corresponding authors.

## Supplementary Materials

The following supporting information can be downloaded at: https://www.mdpi.com/article/10.3390/ph17060781/s1, Figure S1. Raman spectra of phenylboronic acid; Figure S2. Raman spectra of β-d-fructopyranoborate (FB-1); Figure S3. Raman spectra comparison of β-d-arabinopyranoborate (AB-1); Figure S4. IR-FT spectrum of β-d-fructopyranoborate (FB-1); Figure S5. IR-FT spectrum of β-d-arabinopyranoborate (AB-1); Figure S6. ^1^H RMN (DMSO-*d*_6_, 300 MHz) of β-d-fructopyranoborate (FB-1); Figure S7. ^13^C RMN (DMSO-*d*_6_, 76.5 MHz) of β-d-fructopyranoborate (FB-1); Figure S8. ^11^B RMN (DMSO-*d*_6_, 96 MHz) of β-d-fructopyranoborate (FB-1); Figure S9. ^1^H RMN (DMSO-*d*_6_, 300 MHz) of β-d-arabinopyranoborate (AB-1); Figure S10. ^13^C RMN (DMSO-*d*_6_, 76.5 MHz) of β-d-arabinopyranoborate (AB-1); Figure S11. ^11^B RMN (DMSO-*d*_6_, 96 MHz) of β-d-arabinopyranoborate (AB-1); Figure S12. Protox II server prediction for β-d-fructopyranoborate (FB-1); Figure S13. Protox II server prediction for β-d-arabinopyranoborate (AB-1); Figure S14. SwissADME server prediction for β-d-fructopyranoborate (FB-1); Figure S15. SwissADME server prediction for β-d-arabinopyranoborate (AB-1); Figure S16. Molinspiration server prediction for β-d-fructopyranoborate (FB-1); Figure S17. Molinspiration server prediction for β-d-arabinopyranoborate (AB-1); Figure S18. SwissTarget server prediction for β-d-fructopyranoborate (FB-1); Figure S19. SwissTarget server prediction for β-d-arabinopyranoborate (AB-1). CIF data of FB-1 and AB-1.
