# Supplementary material for: One-Step Synthesis, Crystallography, and Acute Toxicity of Two Boron–Carbohydrate Adducts That Induce Sedation in Mice
Source: Pharmaceuticals (Basel). 2024 Jun 14;17(6):781. doi: 10.3390/ph17060781 (PMC11206247; doi:10.3390/ph17060781)

One-step synthesis, crystallography, and acute toxicity of two boronic carbohydrate adducts which induced sedation in mice.

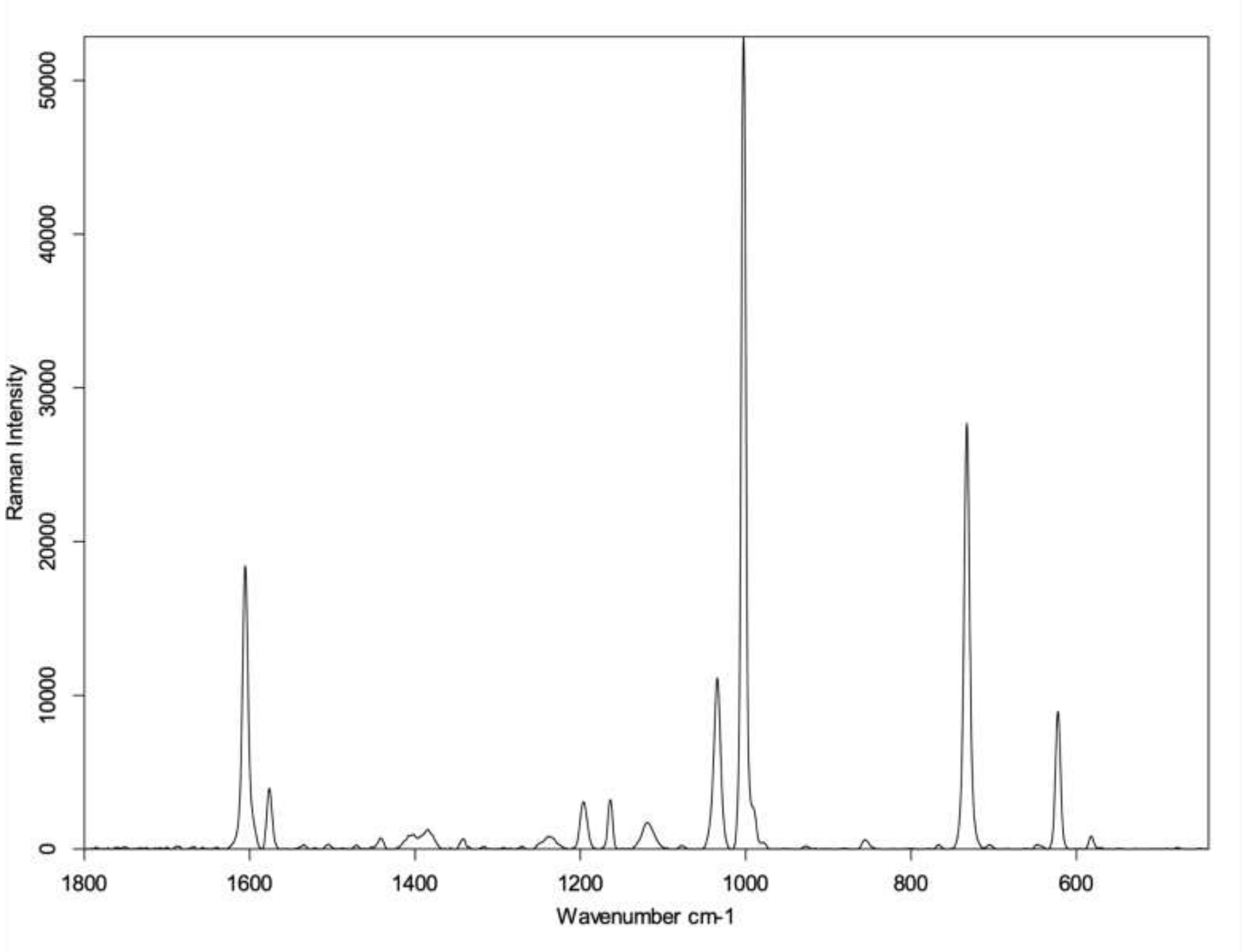

Fig. S1. Raman spectra of phenylboronic acid.

Supplementary materials

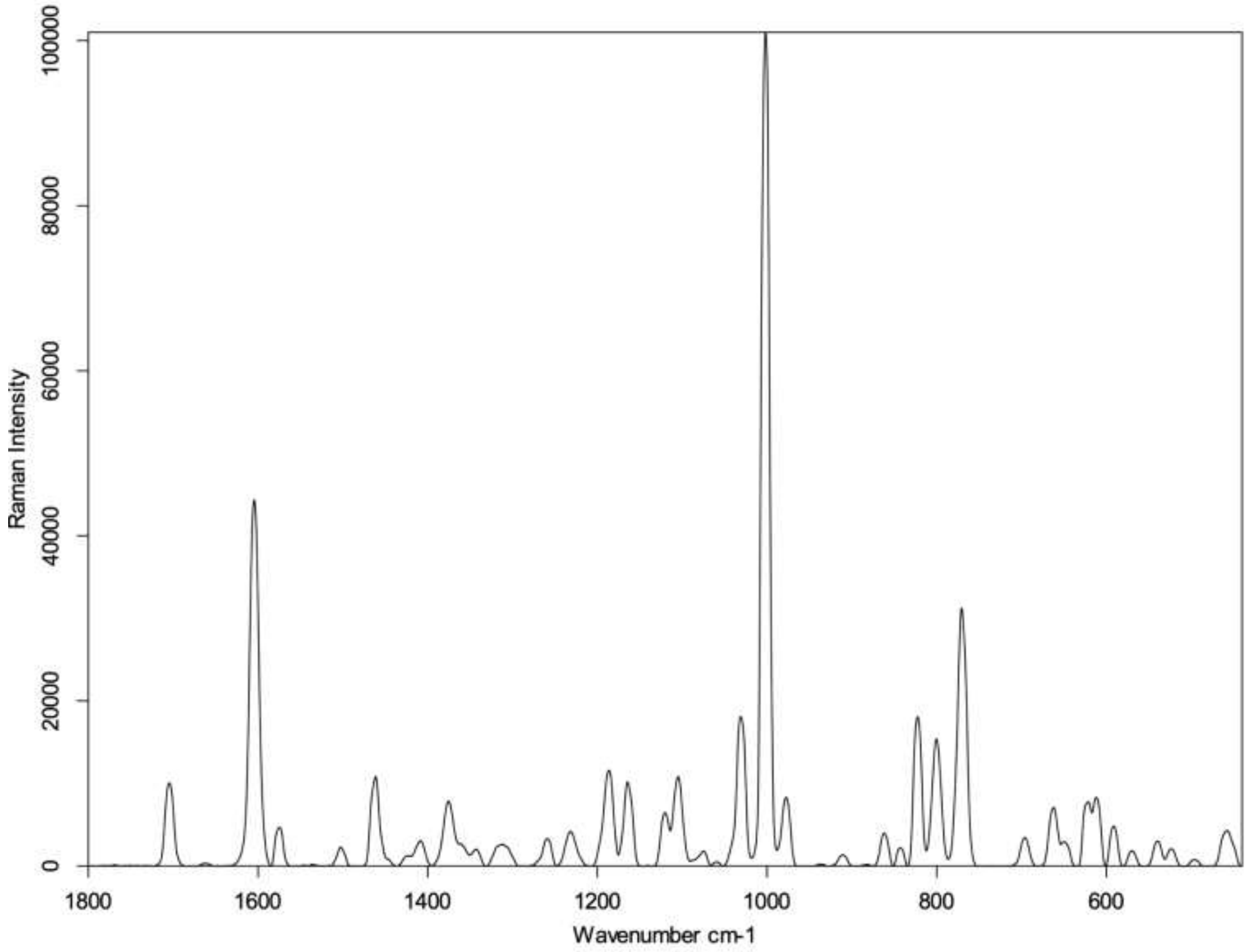

Fig. S2. Raman spectra of β-D-fructopyranoborate (FB-1).

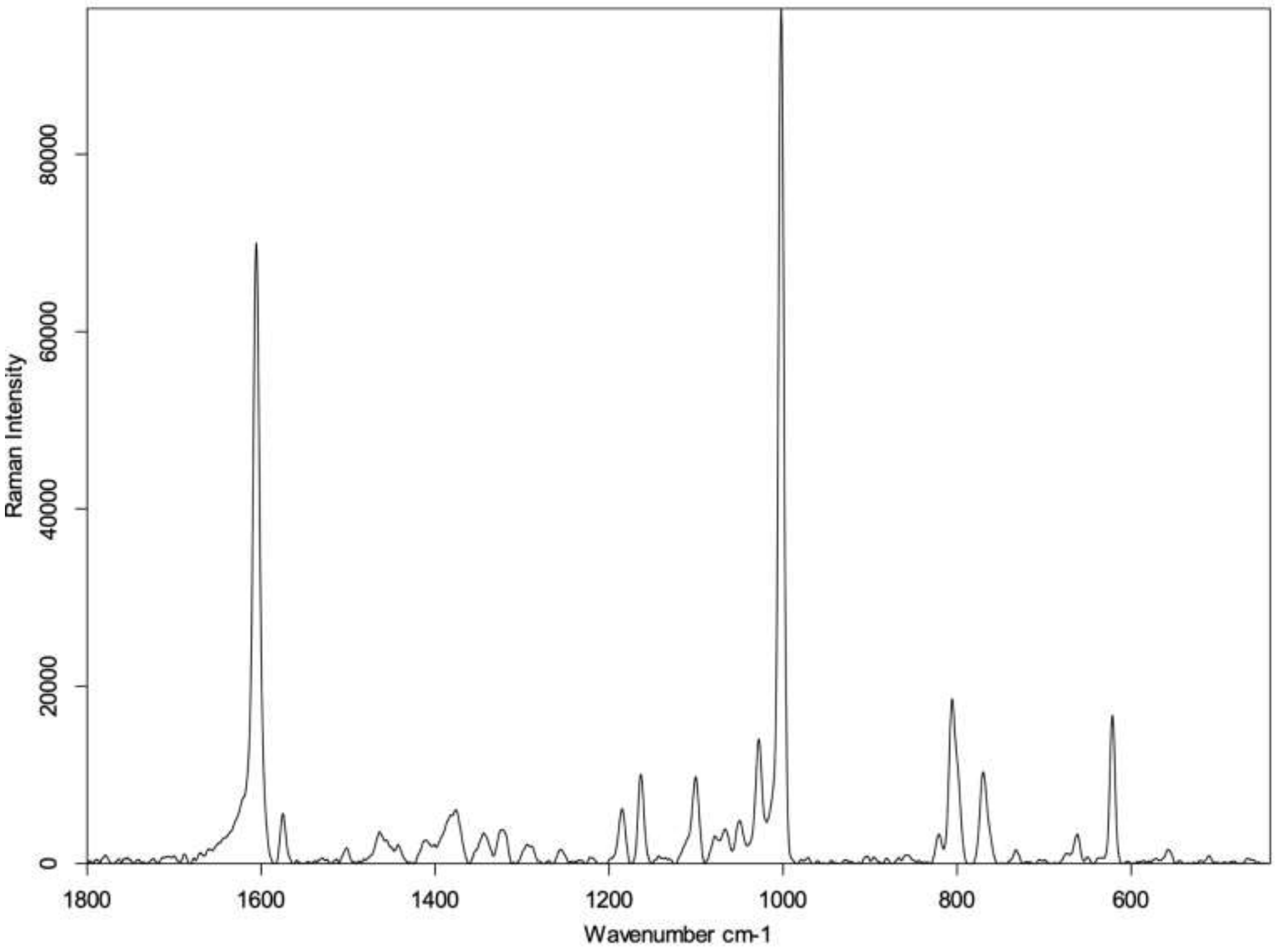

Fig. S3. Raman spectra of β-D-arabinopyranoborate (AB-1).

One-step synthesis, crystallography, and acute toxicity of two boronic carbohydrate adducts which induced sedation in mice.

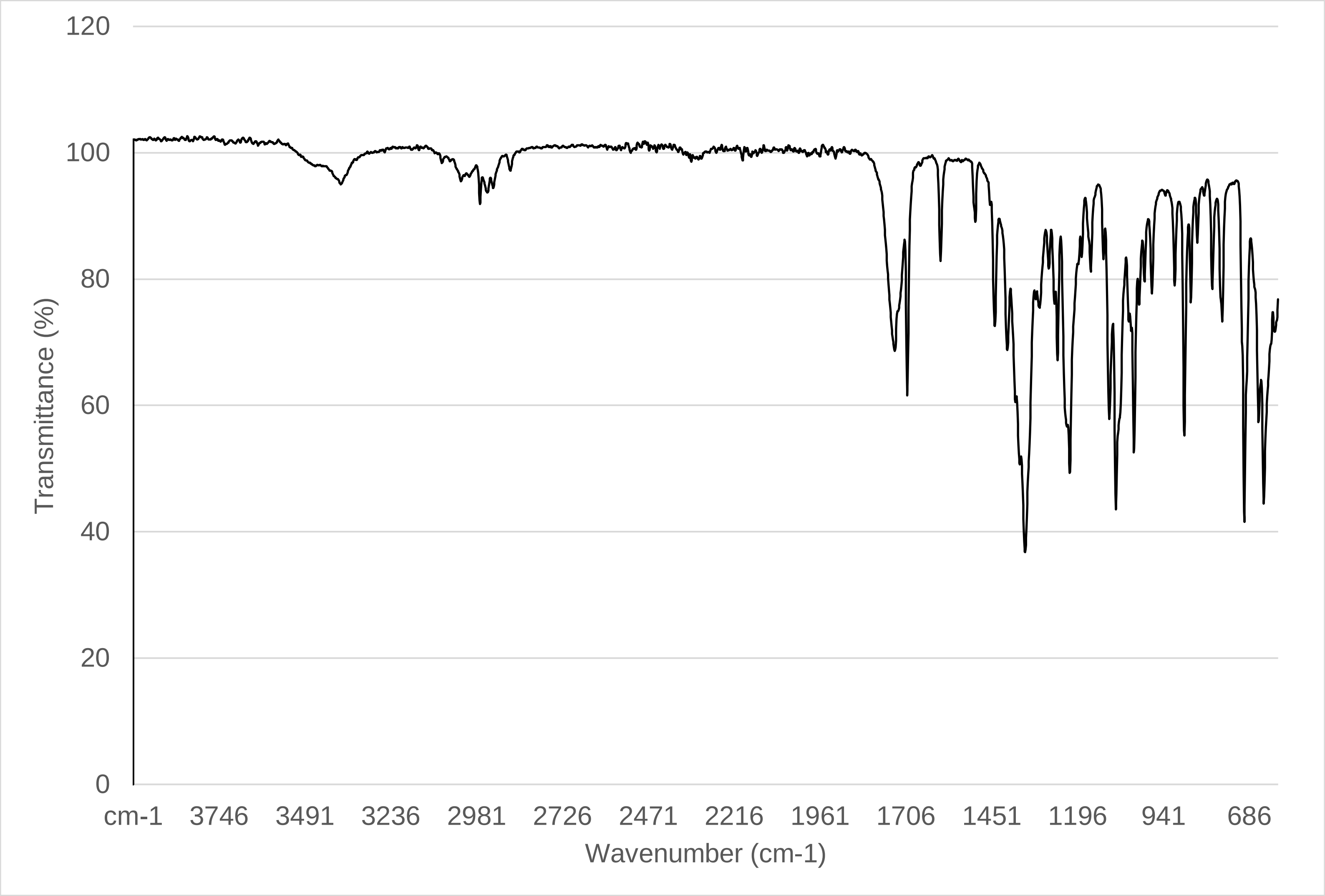

Fig. S4. IR-FT spectrum of the β-D-fructopyranoborate (FB-1).

Supplementary materials

One-step synthesis, crystallography, and acute toxicity of two boronic carbohydrate adducts which induced sedation in mice.

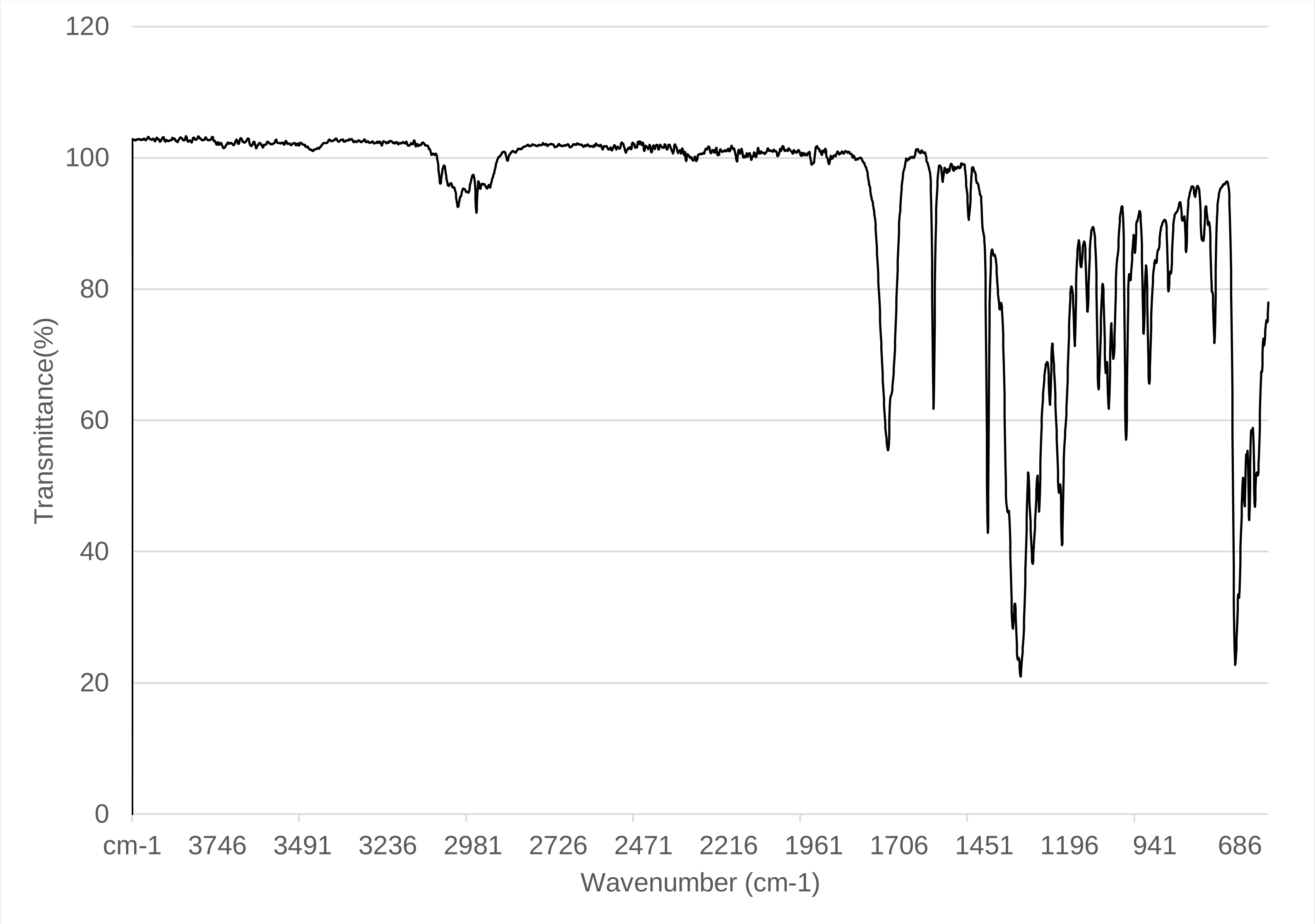

Fig. S5. IR-FT spectrum of the β-D-arabinopyranoborate (AB-1).

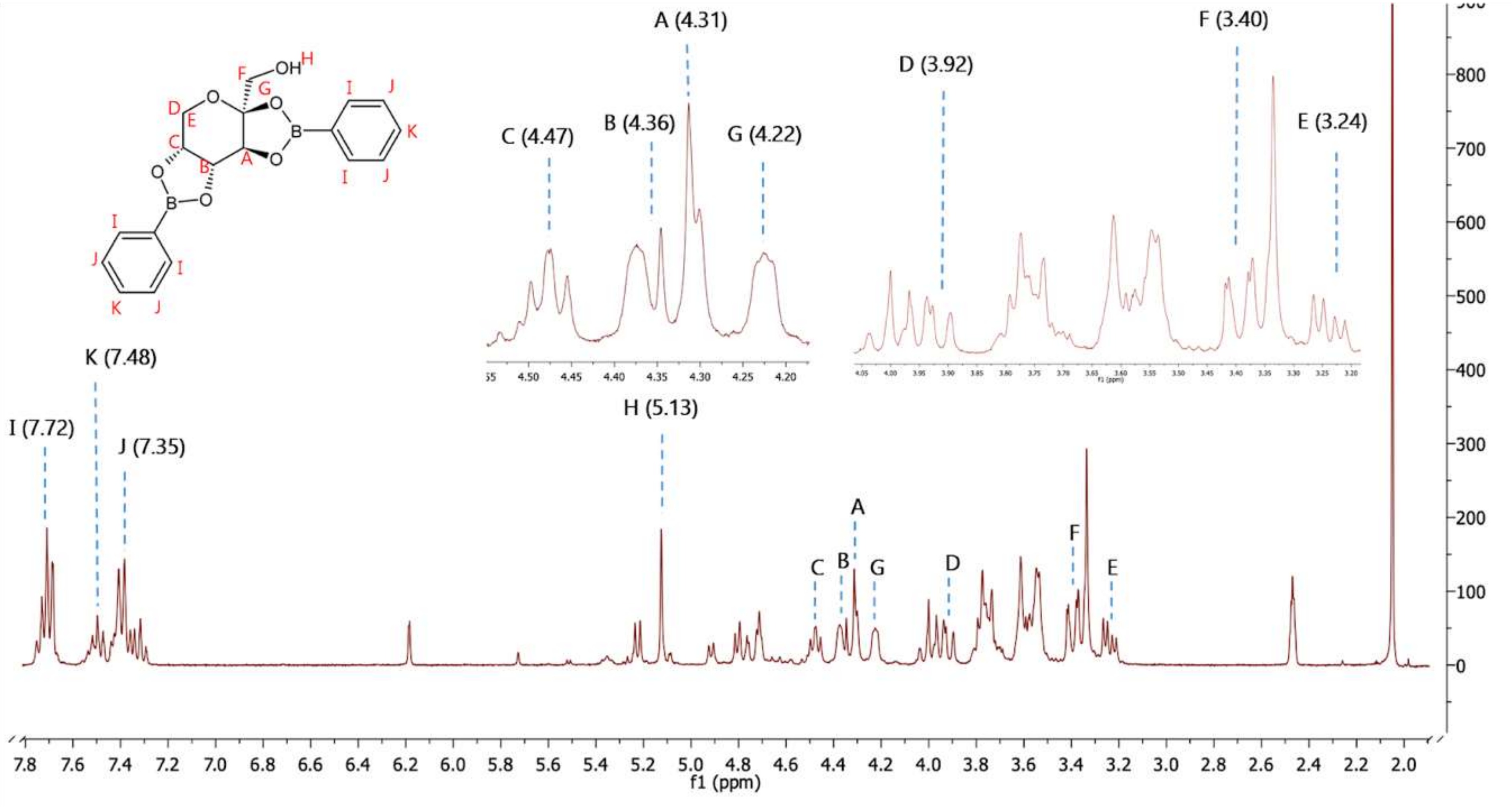

Fig. S6. ^{1}H RMN (DMSO-d_6, 300 MHz) of the β-D-fructopyranoborate (FB-1).

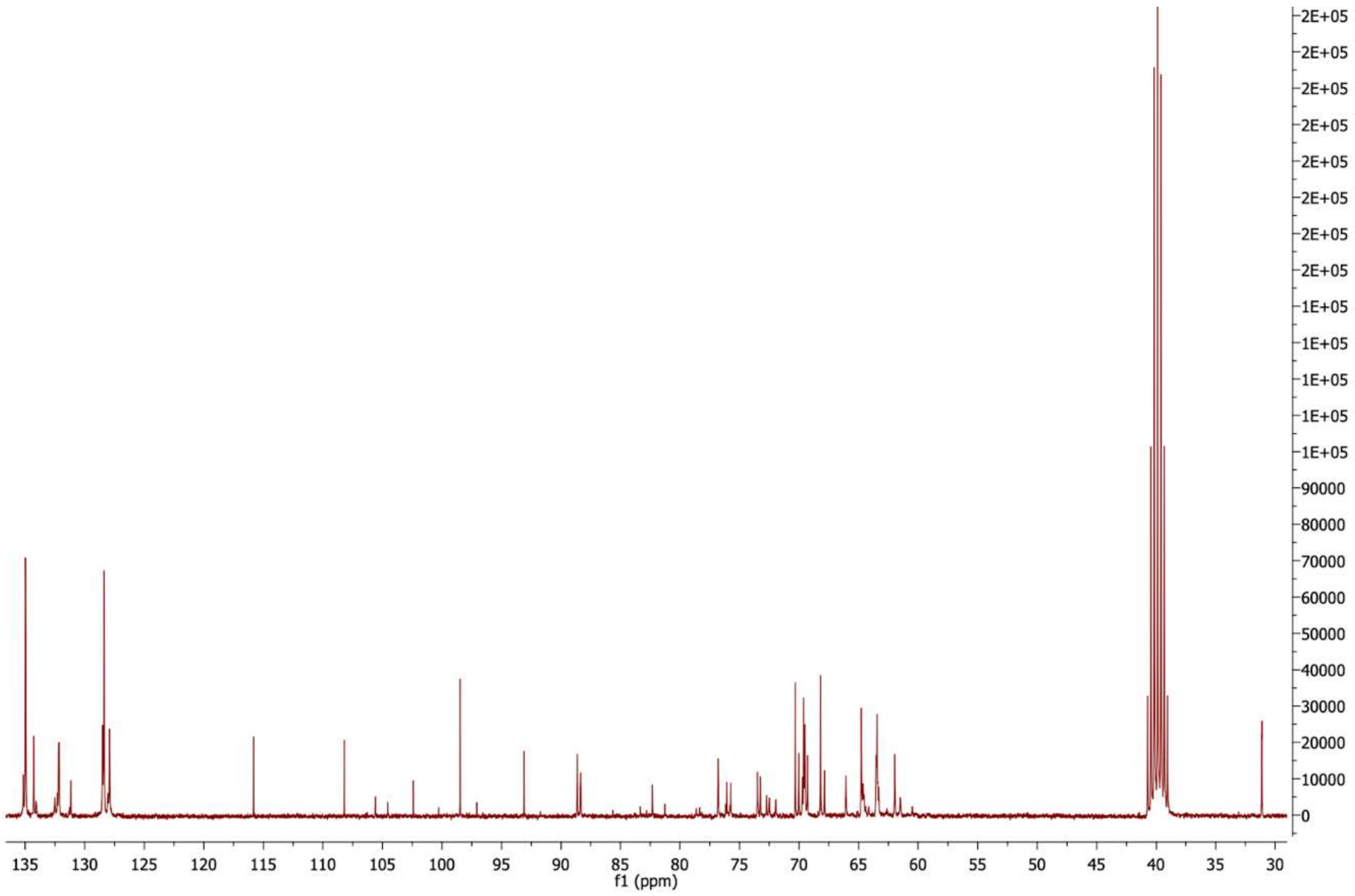

Fig S7. ^{13}C RMN (DMSO-d_6, 76.5 MHz) of the β-D-fructopyranoborate (FB-1).

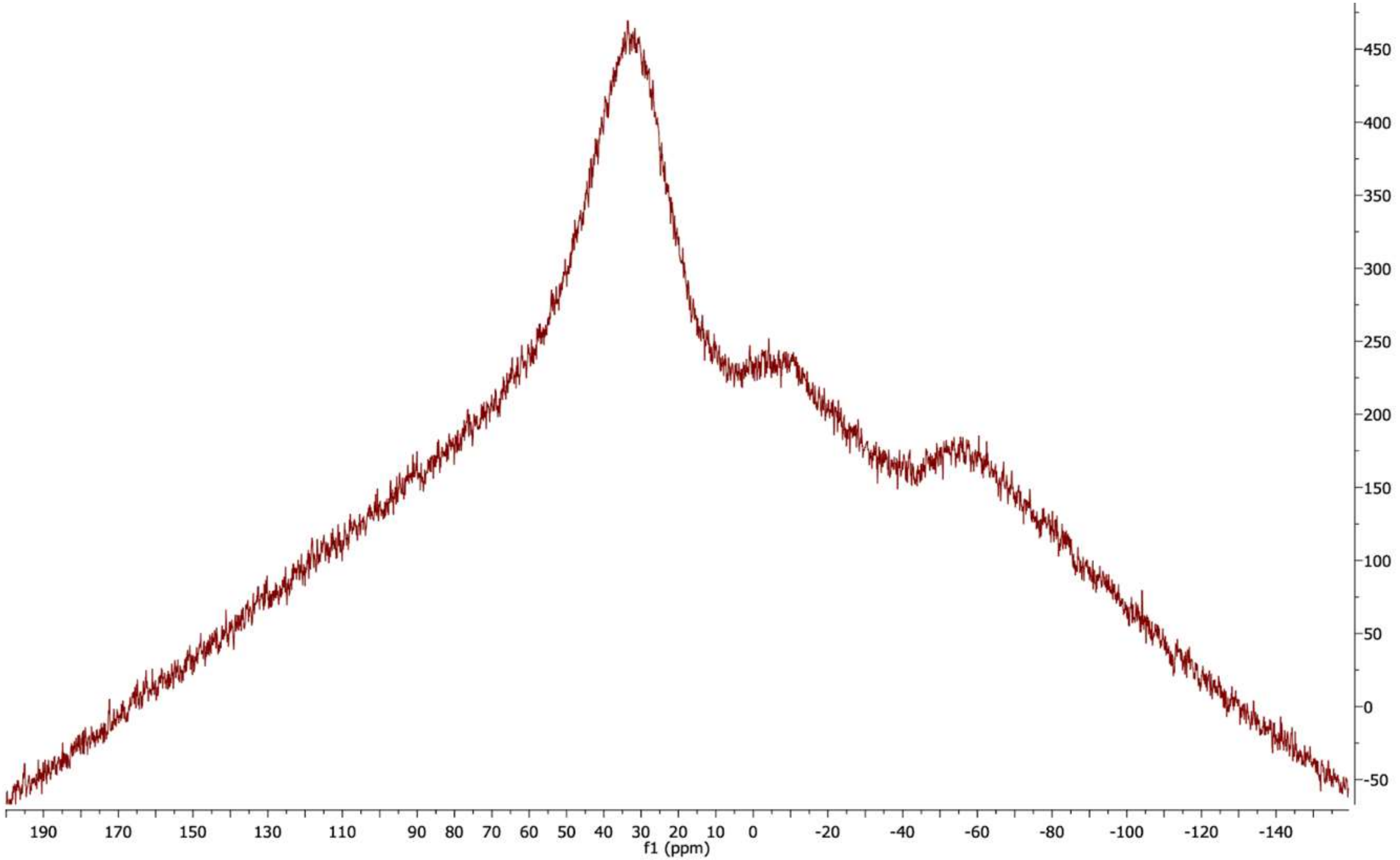

Fig S8. ^{11}B RMN (DMSO-d_6, 96 MHz) of the β-D-fructopyranoborate (FB-1).

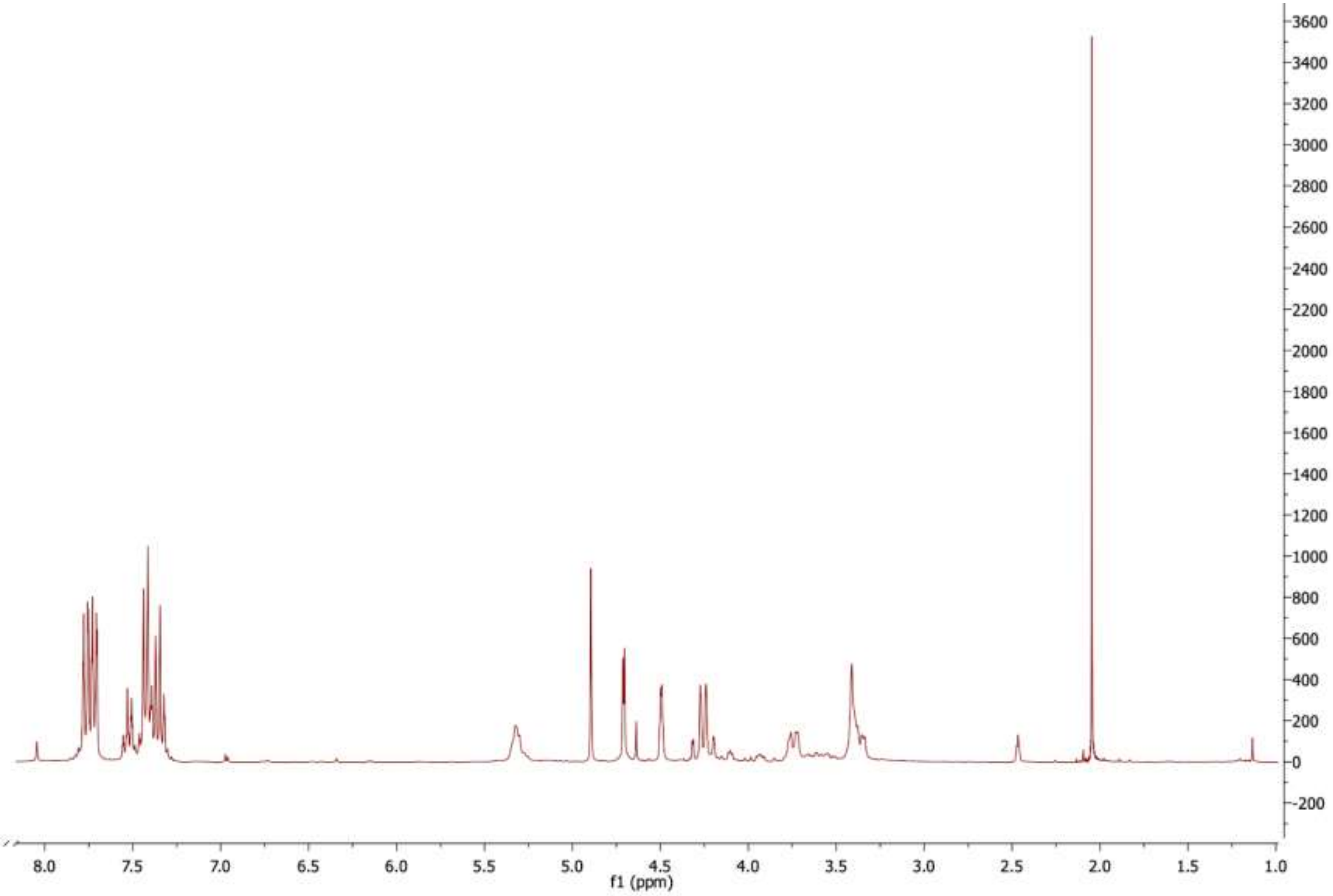

Fig S9. ^{1}H RMN (DMSO-d_6, 300 MHz) of the β-D-arabinopyranoborate (AB-1).

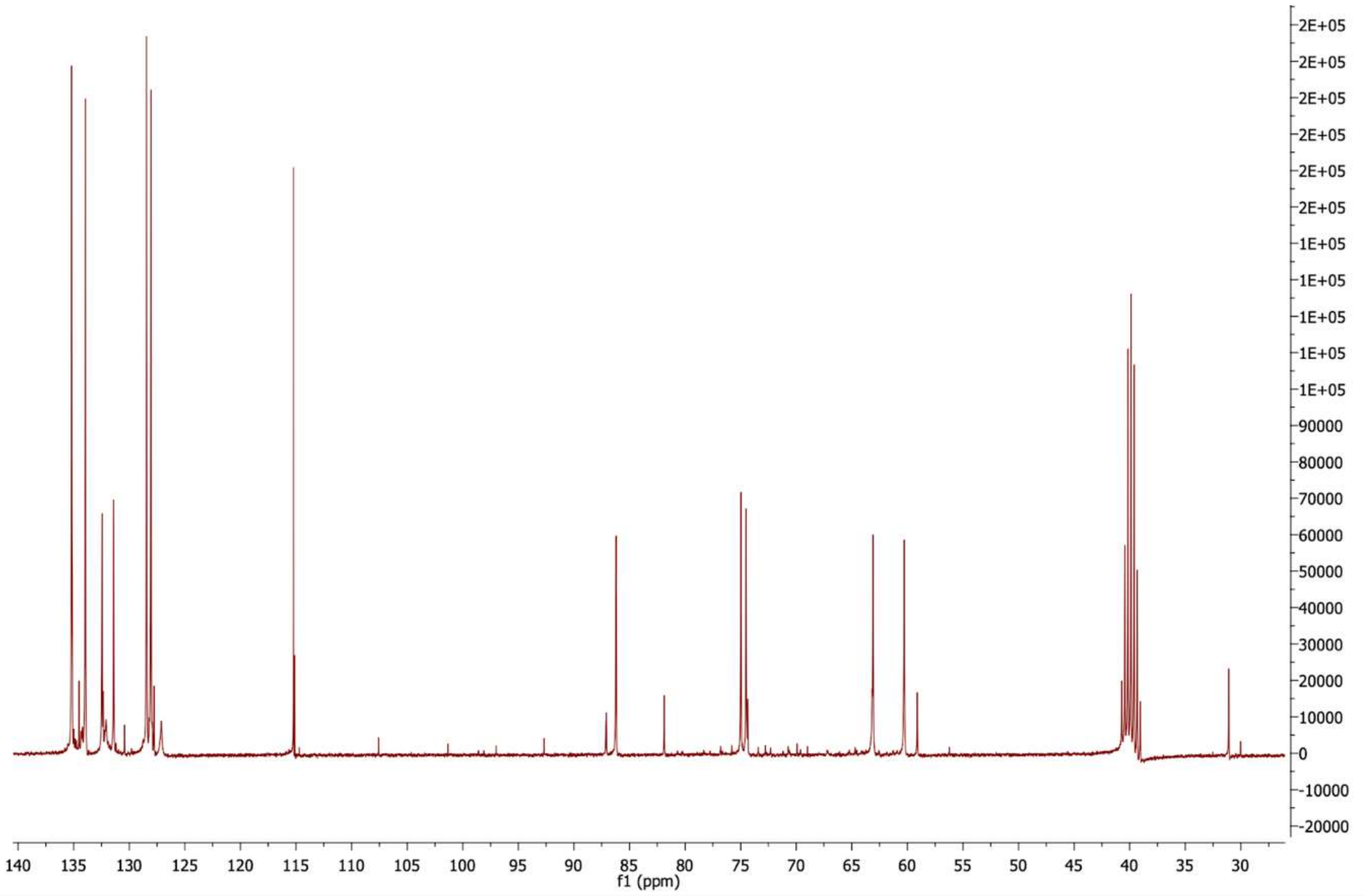

Fig S10. ${}^{13}C$ RMN (DMSO-d_6, 76.5 MHz) of the β-D-arabinopyranoborate (AB-1).

One-step synthesis, crystallography, and acute toxicity of two boronic carbohydrate adducts which induced sedation in mice.

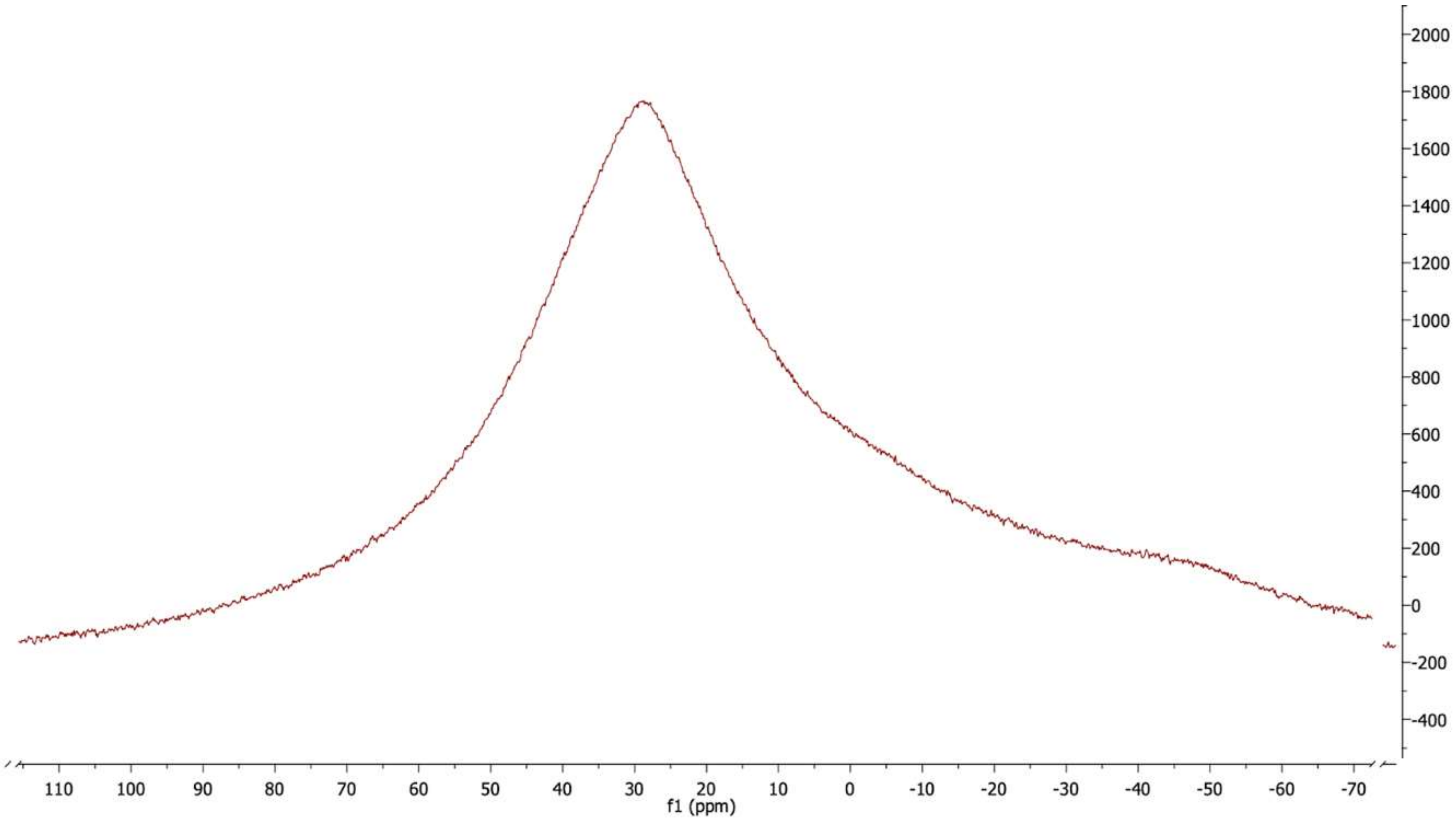

Fig. S11. ^{11}B RMN (DMSO-d_6, 96 MHz) of the β-D-arabinopyranoborate (AB-1).

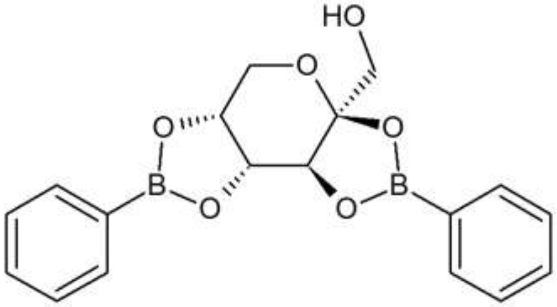

Predicted LD50: 1130mg/kg

Predicted Toxicity Class: 4

1	2	3	4	5	6

Average similarity: 40.08%

Prediction accuracy: 54.26%

Name	FB-1
Molweight	351.96
Number of hydrogen bond acceptors	8
Number of hydrogen bond donors	1
Number of atoms	26
Number of bonds	30
Number of rotable bonds	3
Molecular refractivity	94.9
Topological Polar Surface Area	66.38
octanol/water partition coefficient (logP)	-0.3

Fig. S12. Prediction in the Protox II server for the β-D-fructopyranoborate (FB-1).

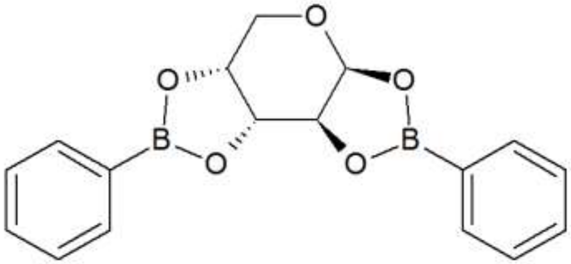

Predicted LD50: 180mg/kg

Predicted Toxicity Class: 3

1	2	3	4	5	6

Average similarity: 39.34%

Prediction accuracy: 23%

Name	AB-1
Molweight	321.93
Number of hydrogen bond acceptors	7
Number of hydrogen bond donors	0
Number of atoms	24
Number of bonds	28
Number of rotable bonds	2
Molecular refractivity	88.89
Topological Polar Surface Area	46.15
octanol/water partition coefficient (logP)	0.33

Fig. S13. Prediction in the Protox II server for the β-D-arabinopyranoborate (AB-1).

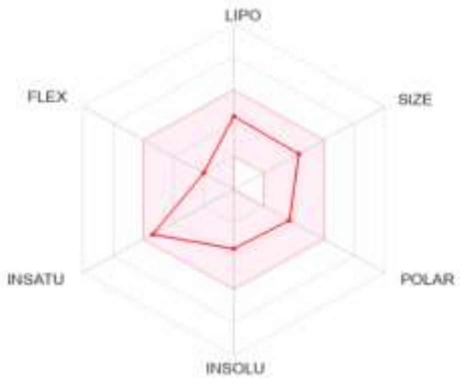

Physicochemical Properties	
Formula	C18H18B2O6
Molecular weight	351.95 g/mol
Num. heavy atoms	26
Num. arom. heavy atoms	12
Fraction Csp3	0.33
Num. rotatable bonds	3
Num. H-bond acceptors	6
Num. H-bond donors	1
Molar Refractivity	94.9
TPSA	66.38 Å²

Lipophilicity	
Log Po/w (iLOGP)	0
Log Po/w (XLOGP3)	2.24
Log Po/w (WLOGP)	-0.3
Log Po/w (MLOGP)	0.04
Log Po/w (SILICOS-IT)	-0.72
Consensus Log Po/w	0.25

Water Solubility	
Log S (ESOL)	-3.58
Solubility	9.32e-02 mg/ml ; 2.65e-04 mol/l
Class	Soluble
Log S (Ali)	-3.27
Solubility	1.89e-01 mg/ml ; 5.37e-04 mol/l
Class	Soluble
Log S (SILICOS-IT)	-4.16
Solubility	2.45e-02 mg/ml ; 6.95e-05 mol/l
Class	Moderately soluble

Pharmacokinetics	
GI absorption	High
BBB permeant	No
P-gp substrate	No
CYP1A2 inhibitor	Yes
CYP2C19 inhibitor	No
CYP2C9 inhibitor	No
CYP2D6 inhibitor	Yes
CYP3A4 inhibitor	No
Log Kp (skin permeation)	-6.86 cm/s

Druglikeness	
Lipinski	Yes; 0 violation
Ghose	Yes
Veber	Yes
Egan	Yes
Muegge	Yes
Bioavailability Score	0.55

Medicinal Chemistry	
PAINS	0 alert
Brenk	1 alert: heavy_metal
Leadlikeness	No; 1 violation: MW>350
Synthetic accessibility	4.88

Fig. S14. Prediction in the SwissADME server for the β-D-fructopyranoborate (FB-1).

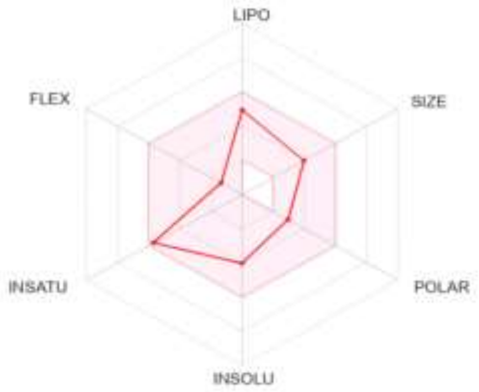

Physicochemical Properties	
Formula	C17H16B2O5
Molecular weight	321.93 g/mol
Num. heavy atoms	24
Num. arom. heavy atoms	12
Fraction Csp3	0.29
Num. rotatable bonds	2
Num. H-bond acceptors	5
Num. H-bond donors	0
Molar Refractivity	88.89
TPSA	46.15 Å²

Lipophilicity	
Log Po/w (iLOGP)	0
Log Po/w (XLOGP3)	3.11
Log Po/w (WLOGP)	0.33
Log Po/w (MLOGP)	0.62
Log Po/w (SILICOS-IT)	-0.56
Consensus Log Po/w	0.7

Water Solubility	
Log S (ESOL)	-4.03
Solubility	2.98e-02 mg/ml ; 9.26e-05 mol/l
Class	Moderately soluble
Log S (Ali)	-3.75
Solubility	5.76e-02 mg/ml ; 1.79e-04 mol/l
Class	Soluble
Log S (SILICOS-IT)	-4.13
Solubility	2.40e-02 mg/ml ; 7.45e-05 mol/l
Class	Moderately soluble

Pharmacokinetics	
GI absorption	High
BBB permeant	No
P-gp substrate	No
CYP1A2 inhibitor	Yes
CYP2C19 inhibitor	No
CYP2C9 inhibitor	No
CYP2D6 inhibitor	Yes
CYP3A4 inhibitor	No
Log Kp (skin permeation)	-6.06 cm/s

Druglikeness	
Lipinski	Yes; 0 violation
Ghose	Yes
Veber	Yes
Egan	Yes
Muegge	Yes
Bioavailability Score	0.55

Medicinal Chemistry	
PAINS	0 alert
Brenk	1 alert: heavy_metal
Leadlikeness	Yes
Synthetic accessibility	4.73

Fig. S15. Prediction in the SwissADME server for the β-D-arabinopyranoborate (AB-1).

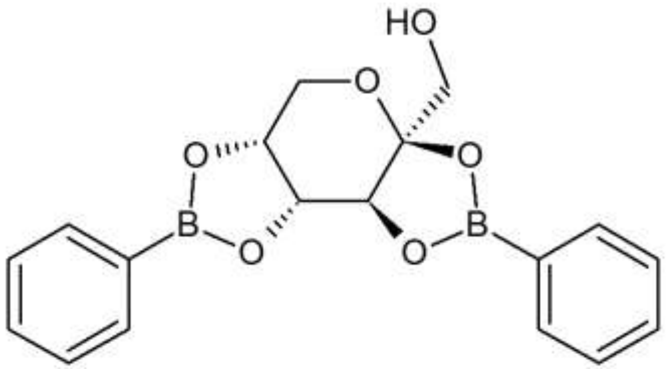

miLogP	1.7
TPSA	66.4
natoms	26
MW	351.96
nON	6
nOHNH	1
nviolations	0
nrotb	3
volume	352.54

Fig. S16. Prediction in the Molinspiration server for the β-D-fructopyranoborate (FB-1).

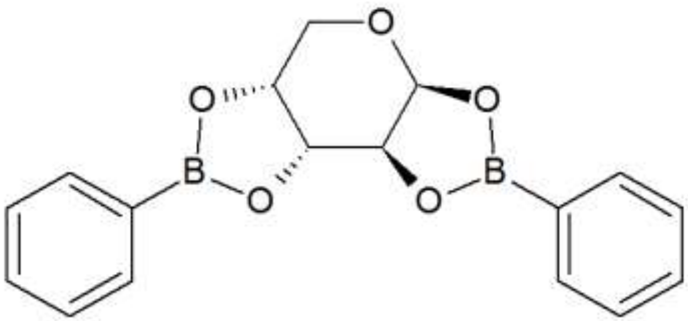

miLogP	2.26
TPSA	46.17
natoms	24
MW	321.93
nON	5
nOHNH	0
nviolations	0
nrotb	2
volume	328.04

Fig. S17. Prediction in the Molinspiration server for the β-D-arabinopyranoborate (AB-1).

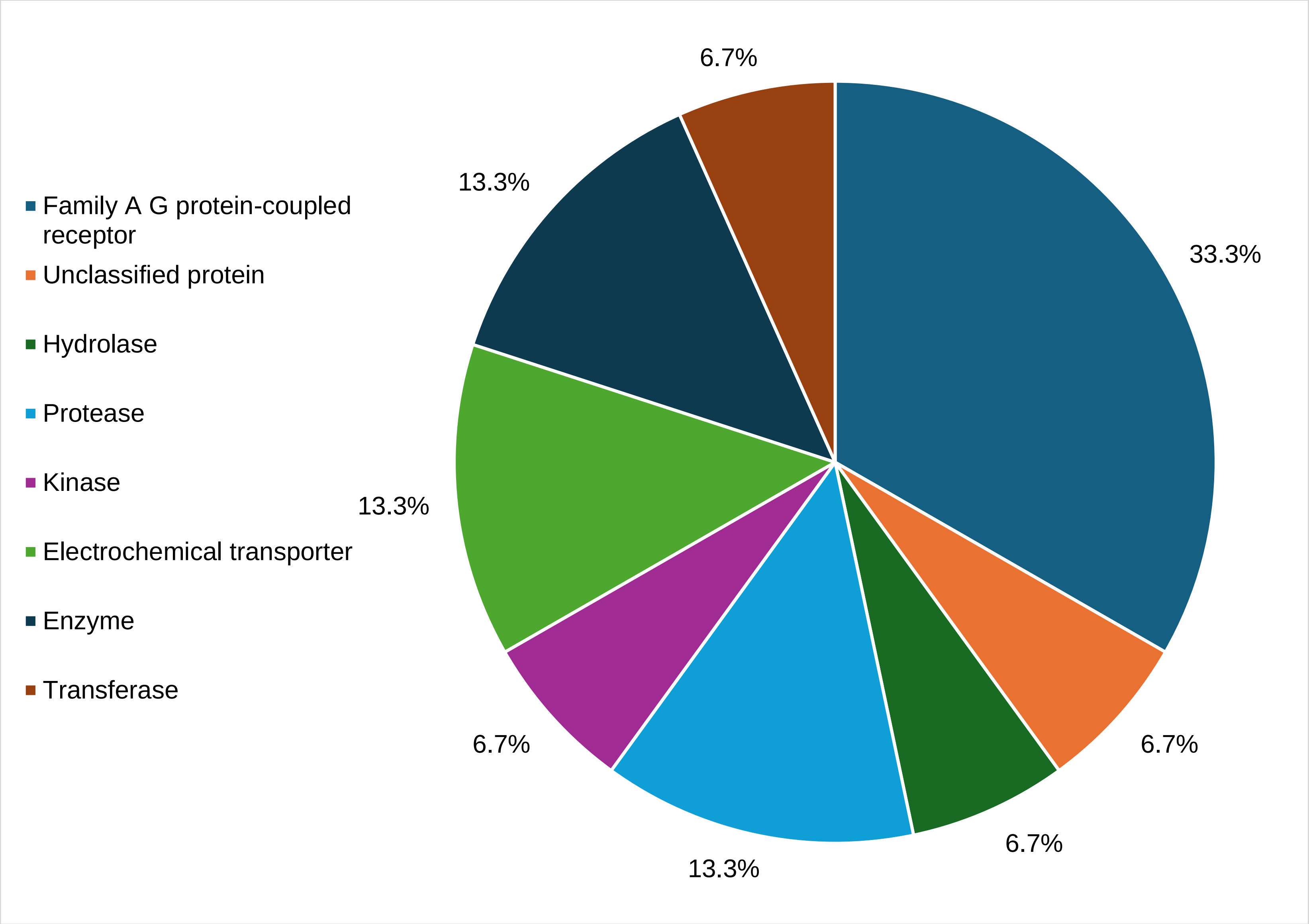

Fig. S18. Prediction in the SwissTarget server for the β-D-fructopyranoborate (FB-1).

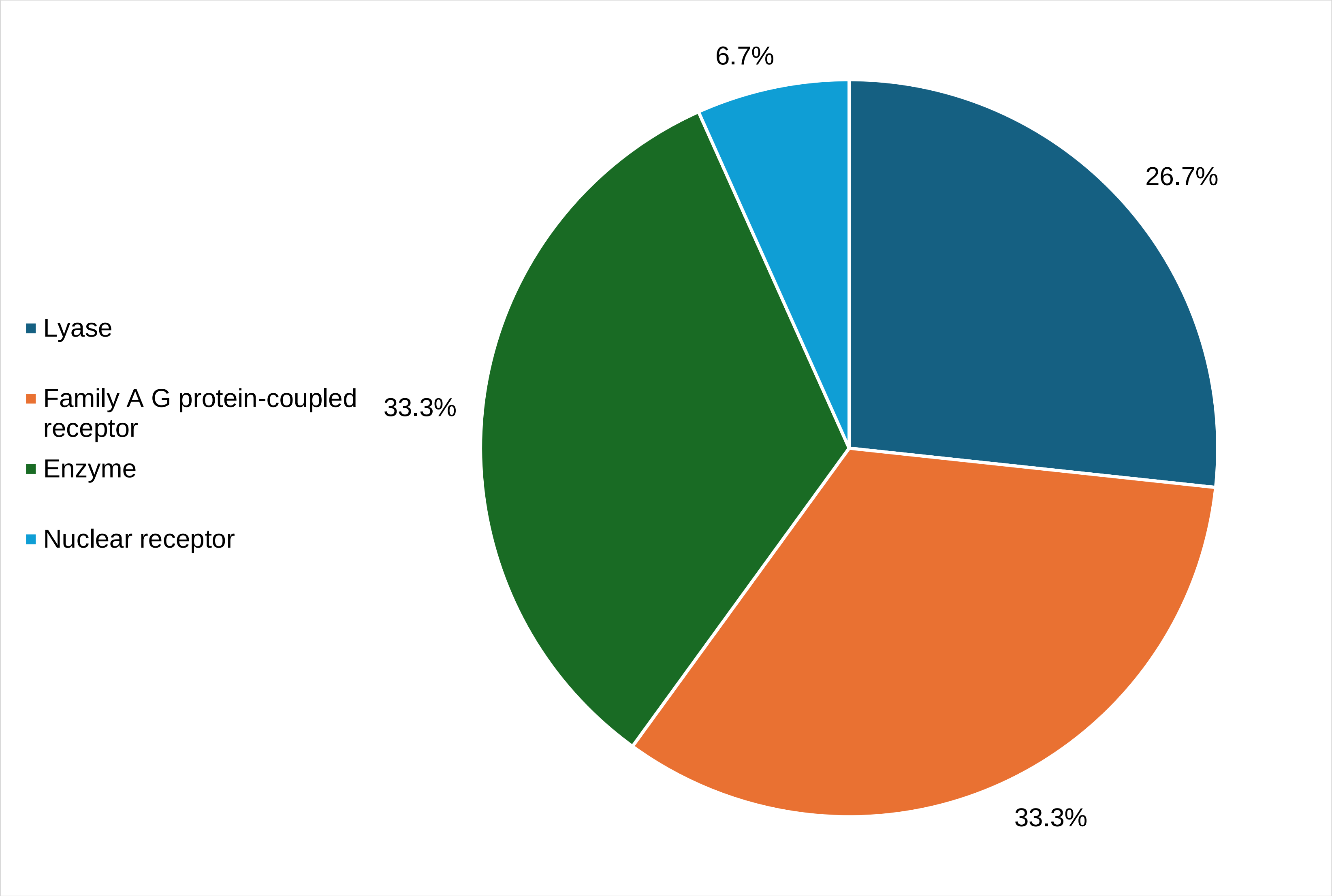

Fig. S19. Prediction in the SwissTarget server for the β-D-arabinopyranoborate (AB-1).

checkCIF/PLATON report

Structure factors have been supplied for datablock(s) shelx

THIS REPORT IS FOR GUIDANCE ONLY. IF USED AS PART OF A REVIEW PROCEDURE FOR PUBLICATION, IT SHOULD NOT REPLACE THE EXPERTISE OF AN EXPERIENCED CRYSTALLOGRAPHIC REFEREE.

No syntax errors found. CIF dictionary Interpreting this report

Datablock: shelx

Bond precision:	C-C = 0.0047 A		Wavelength=0.71073
Cell:	a=7.9885(5)	b=10.6053(5)	c=24.9565(15)
	alpha=90	beta=90	gamma=90
Temperature:	173 K		

	Calculated	Reported
Volume	2114.3(2)	2114.3(2)
Space group	P 21 21 21	P 21 21 21
Hall group	P 2ac 2ab	P 2ac 2ab
Moiety formula	C18 H18 B2 O6, C3 H6 O	?
Sum formula	C21 H24 B2 O7	C10.50 H12 B O3.50
Mr	410.02	205.01
Dx,g cm-3	1.288	1.288
Z	4	8
Mu (mm-1)	0.094	0.094
F000	864.0	864.0
F000'	864.48	
h,k,lmax	9,12,30	9,12,30
Nref	3859[2228]	3843
Tmin,Tmax		0.861,0.862
Tmin'		

Correction method= # Reported T Limits: Tmin=0.861 Tmax=0.862
AbsCorr = SPHERE

Data completeness= 1.72/1.00 Theta(max)= 25.323

R(reflections)= 0.0365(2826) wR2(reflections)= 0.0982(3843)

S = 1.014 Npar= 274

The following ALERTS were generated. Each ALERT has the format
test-name_ALERT_alert-type_alert-level.
Click on the hyperlinks for more details of the test.

Alert level C

```
PLAT244_ALERT_4_C Low    'Solvent' Ueq as Compared to Neighbors of        C21 Check
PLAT340_ALERT_3_C Low Bond Precision on  C-C Bonds ...............    0.00468 Ang.
PLAT767_ALERT_4_C INS Embedded LIST 6 Instruction Should be LIST 4     Please Check
PLAT911_ALERT_3_C Missing FCF Refl Between Thmin & STh/L=    0.600          9 Report
                0 10  0,   3 12  0,   0 10  1,   1  0  2,   0 10  2,   0 10  3,
                2  0  6,   0 11 12,   0  0 22,
PLAT934_ALERT_3_C Number of (Iobs-Icalc)/Sigma(W) > 10 Outliers ..          1 Check
                3  0 11,
```

Alert level G

```
PLAT007_ALERT_5_G Number of Unrefined Donor-H Atoms .............          1 Report
              H19
PLAT032_ALERT_4_G Std. Uncertainty on Flack Parameter Value High .      0.400 Report
PLAT045_ALERT_1_G Calculated and Reported Z Differ by a Factor ...      0.500 Check
PLAT395_ALERT_2_G Deviating  X-O-Y   Angle From 120 for O3       .      108.0 Degree
PLAT395_ALERT_2_G Deviating  X-O-Y   Angle From 120 for O4       .      108.2 Degree
PLAT395_ALERT_2_G Deviating  X-O-Y   Angle From 120 for O5       .      107.7 Degree
PLAT395_ALERT_2_G Deviating  X-O-Y   Angle From 120 for O6       .      109.2 Degree
PLAT791_ALERT_4_G Model has Chirality at C3         (Sohncke SpGr)          R Verify
PLAT791_ALERT_4_G Model has Chirality at C4         (Sohncke SpGr)          R Verify
PLAT791_ALERT_4_G Model has Chirality at C5         (Sohncke SpGr)          S Verify
PLAT791_ALERT_4_G Model has Chirality at C6         (Sohncke SpGr)          R Verify
PLAT883_ALERT_1_G No Info/Value for _atom_sites_solution_primary .     Please Do !
PLAT899_ALERT_4_G SHELXL2018 is Deprecated and Succeeded by SHELXL     2019/3 Note
PLAT910_ALERT_3_G Missing # of FCF Reflection(s) Below Theta(Min).          1 Note
                0  0  2,
PLAT969_ALERT_5_G The 'Henn et al.' R-Factor-gap value ..........      2.485 Note
              Predicted wR2: Based on SigI**2  3.95 or SHELX Weight  9.69
PLAT978_ALERT_2_G Number C-C Bonds with Positive Residual Density.          0 Info
```

0 **ALERT level A** = Most likely a serious problem - resolve or explain
0 **ALERT level B** = A potentially serious problem, consider carefully
5 **ALERT level C** = Check. Ensure it is not caused by an omission or oversight
16 **ALERT level G** = General information/check it is not something unexpected

2 ALERT type 1 CIF construction/syntax error, inconsistent or missing data
5 ALERT type 2 Indicator that the structure model may be wrong or deficient
4 ALERT type 3 Indicator that the structure quality may be low
8 ALERT type 4 Improvement, methodology, query or suggestion
2 ALERT type 5 Informative message, check

Validation response form

Please find below a validation response form (VRF) that can be filled in and pasted into your CIF.

```
# start Validation Reply Form
_vrf_PLAT244_shelx
;
PROBLEM: Low    'Solvent' Ueq as Compared to Neighbors of        C21 Check
RESPONSE: ...
;
_vrf_PLAT340_shelx
;
PROBLEM: Low Bond Precision on  C-C Bonds ...............    0.00468 Ang.
RESPONSE: ...
;
_vrf_PLAT767_shelx
;
PROBLEM: INS Embedded LIST 6 Instruction Should be LIST 4     Please Check
RESPONSE: ...
;
_vrf_PLAT911_shelx
;
PROBLEM: Missing FCF Refl Between Thmin & STh/L=    0.600          9 Report
RESPONSE: ...
;
_vrf_PLAT934_shelx
;
PROBLEM: Number of (Iobs-Icalc)/Sigma(W) > 10 Outliers ..          1 Check
RESPONSE: ...
;
# end Validation Reply Form
```

It is advisable to attempt to resolve as many as possible of the alerts in all categories. Often the minor alerts point to easily fixed oversights, errors and omissions in your CIF or refinement strategy, so attention to these fine details can be worthwhile. In order to resolve some of the more serious problems it may be necessary to carry out additional measurements or structure refinements. However, the purpose of your study may justify the reported deviations and the more serious of these should normally be commented upon in the discussion or experimental section of a paper or in the "special_details" fields of the CIF. checkCIF was carefully designed to identify outliers and unusual parameters, but every test has its limitations and alerts that are not important in a particular case may appear. Conversely, the absence of alerts does not guarantee there are no aspects of the results needing attention. It is up to the individual to critically assess their own results and, if necessary, seek expert advice.

Publication of your CIF in IUCr journals

A basic structural check has been run on your CIF. These basic checks will be run on all CIFs submitted for publication in IUCr journals (*Acta Crystallographica*, *Journal of Applied Crystallography*, *Journal of Synchrotron Radiation*); however, if you intend to submit to *Acta Crystallographica Section C* or *E* or *IUCrData*, you should make sure that full publication checks are run on the final version of your CIF prior to submission.

Publication of your CIF in other journals

Please refer to the *Notes for Authors* of the relevant journal for any special instructions relating to CIF submission.

PLATON version of 13/05/2024; check.def file version of 04/05/2024

Datablock shelx - ellipsoid plot

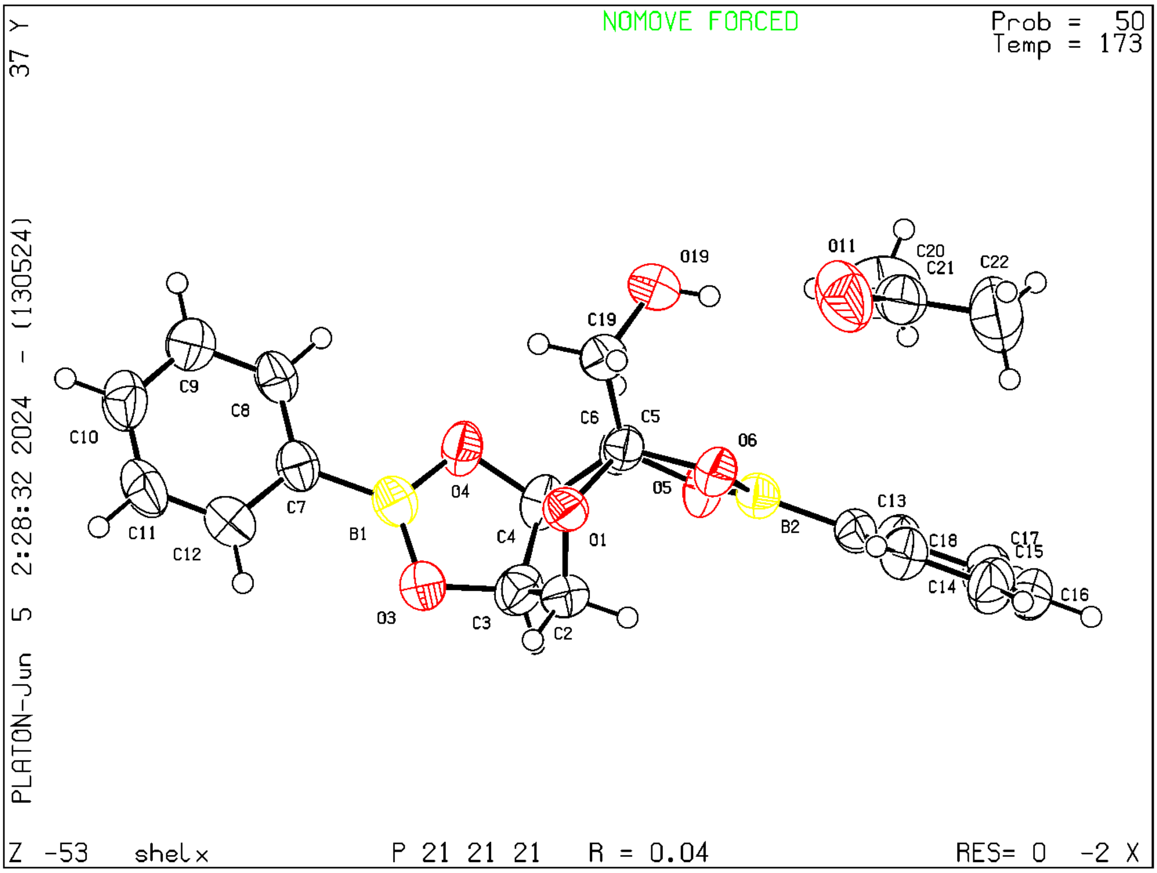

checkCIF/PLATON report

Structure factors have been supplied for datablock(s) shelx

THIS REPORT IS FOR GUIDANCE ONLY. IF USED AS PART OF A REVIEW PROCEDURE FOR PUBLICATION, IT SHOULD NOT REPLACE THE EXPERTISE OF AN EXPERIENCED CRYSTALLOGRAPHIC REFEREE.

No syntax errors found. CIF dictionary Interpreting this report

Datablock: shelx

```
Bond precision:   C-C = 0.0096 A          Wavelength=0.71073

Cell:              a=9.3975(6)      b=10.1227(6)      c=17.1597(10)
                   alpha=90         beta=90           gamma=90
Temperature:       273 K

                Calculated                     Reported
Volume          1632.37(17)                    1632.37(17)
Space group     P 21 21 21                     P 21 21 21
Hall group      P 2ac 2ab                      P 2ac 2ab
Moiety formula  C17 H16 B2 O5                  C17 H16 B2 O5
Sum formula     C17 H16 B2 O5                  C17 H16 B2 O5
Mr              321.92                         321.92
Dx,g cm-3       1.310                          1.310
Z               4                              4
Mu (mm-1)       0.093                          0.093
F000            672.0                          672.0
F000'           672.37
h,k,lmax        11,12,20                       11,12,20
Nref            2875[ 1667]                    2822
Tmin,Tmax                                      0.861,0.862
Tmin'

Correction method= # Reported T Limits: Tmin=0.861 Tmax=0.862
AbsCorr = SPHERE

Data completeness= 1.69/0.98        Theta(max)= 24.998

                                                   wR2(reflections)=
R(reflections)= 0.0651( 2022)                      0.2241( 2822)

S = 1.240                   Npar= 218
```

The following ALERTS were generated. Each ALERT has the format
test-name_ALERT_alert-type_alert-level.
Click on the hyperlinks for more details of the test.

Alert level C

```
PLAT340_ALERT_3_C Low Bond Precision on  C-C Bonds ...............    0.00963 Ang.
PLAT905_ALERT_3_C Negative K value in the Analysis of Variance ...     -1.456 Report
PLAT911_ALERT_3_C Missing FCF Refl Between Thmin & STh/L=    0.595         26 Report
```

Alert level G

```
PLAT032_ALERT_4_G Std. Uncertainty on Flack Parameter Value High .      0.300 Report
PLAT199_ALERT_1_G Reported _cell_measurement_temperature ..... (K)        273 Check
PLAT200_ALERT_1_G Reported   _diffrn_ambient_temperature ..... (K)        273 Check
PLAT395_ALERT_2_G Deviating  X-O-Y   Angle From 120 for O1       .      108.8 Degree
PLAT395_ALERT_2_G Deviating  X-O-Y   Angle From 120 for O2       .      107.8 Degree
PLAT395_ALERT_2_G Deviating  X-O-Y   Angle From 120 for O3       .      109.3 Degree
PLAT395_ALERT_2_G Deviating  X-O-Y   Angle From 120 for O5       .      108.7 Degree
PLAT883_ALERT_1_G No Info/Value for _atom_sites_solution_primary .     Please Do !
PLAT899_ALERT_4_G SHELXL2018 is Deprecated and Succeeded by SHELXL     2019/3 Note
PLAT933_ALERT_2_G Number of HKL-OMIT Records in Embedded .res File         58 Note
PLAT965_ALERT_2_G The SHELXL WEIGHT Optimisation has not Converged     Please Check
PLAT967_ALERT_5_G Note: Two-Theta Cutoff Value in Embedded .res ..       50.0 Degree
PLAT978_ALERT_2_G Number C-C Bonds with Positive Residual Density.          0 Info
```

```
   0 ALERT level A = Most likely a serious problem - resolve or explain
   0 ALERT level B = A potentially serious problem, consider carefully
   3 ALERT level C = Check. Ensure it is not caused by an omission or oversight
  13 ALERT level G = General information/check it is not something unexpected

   3 ALERT type 1 CIF construction/syntax error, inconsistent or missing data
   7 ALERT type 2 Indicator that the structure model may be wrong or deficient
   3 ALERT type 3 Indicator that the structure quality may be low
   2 ALERT type 4 Improvement, methodology, query or suggestion
   1 ALERT type 5 Informative message, check
```

It is advisable to attempt to resolve as many as possible of the alerts in all categories. Often the minor alerts point to easily fixed oversights, errors and omissions in your CIF or refinement strategy, so attention to these fine details can be worthwhile. In order to resolve some of the more serious problems it may be necessary to carry out additional measurements or structure refinements. However, the purpose of your study may justify the reported deviations and the more serious of these should normally be commented upon in the discussion or experimental section of a paper or in the "special_details" fields of the CIF. checkCIF was carefully designed to identify outliers and unusual parameters, but every test has its limitations and alerts that are not important in a particular case may appear. Conversely, the absence of alerts does not guarantee there are no aspects of the results needing attention. It is up to the individual to critically assess their own results and, if necessary, seek expert advice.

Publication of your CIF in IUCr journals

A basic structural check has been run on your CIF. These basic checks will be run on all CIFs submitted for publication in IUCr journals (*Acta Crystallographica*, *Journal of Applied Crystallography*, *Journal of Synchrotron Radiation*); however, if you intend to submit to *Acta Crystallographica Section C* or *E* or *IUCrData*, you should make sure that full publication checks are run on the final version of your CIF prior to submission.

Publication of your CIF in other journals

Please refer to the *Notes for Authors* of the relevant journal for any special instructions relating to CIF submission.

PLATON version of 06/07/2023; check.def file version of 30/06/2023

Datablock shelx - ellipsoid plot

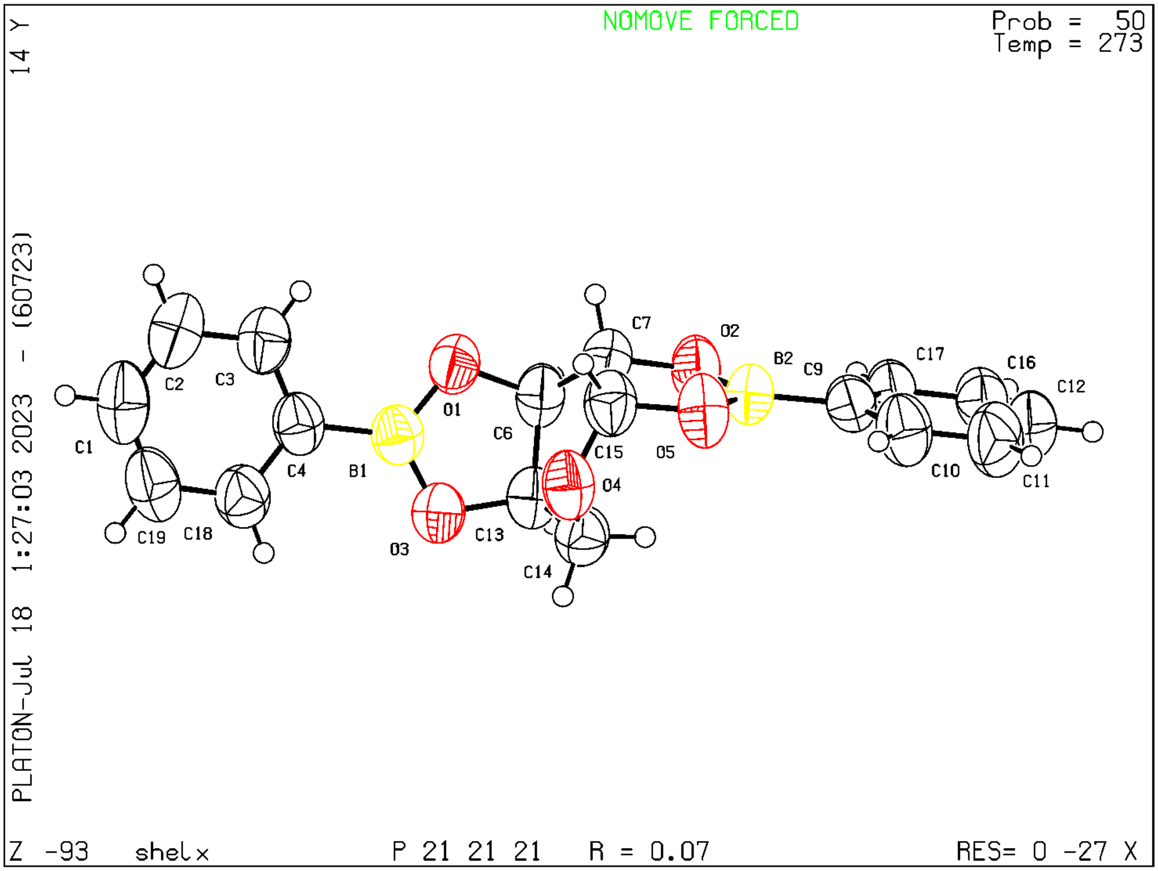

Supplement: Supplementary file 1 [file pharmaceuticals-17-00781-s001.zip › pharmaceuticals-3042313-supplementary.pdf]
